# Bottom‐up Synthesis of Water‐Soluble Gold Nanoparticles Stabilized by N‐Heterocyclic Carbenes: From Structural Characterization to Applications

**DOI:** 10.1002/chem.202201575

**Published:** 2022-08-10

**Authors:** Sophie R. Thomas, Wenjie Yang, David J. Morgan, Thomas E. Davies, Jiao Jiao Li, Roland A. Fischer, Jun Huang, Nikolaos Dimitratos, Angela Casini

**Affiliations:** ^1^ Chair of Medicinal and Bioinorganic Chemistry Department of Chemistry Technical University of Munich Lichtenbergstrasse 4 85747 Garching Germany; ^2^ School of Chemical and Biomolecular Engineering University of Sydney NSW 2006 Australia; ^3^ School of Chemistry Cardiff University Main Building, Park Place Cardiff CF10 3AT U.K.; ^4^ Kolling Institute Faculty of Medicine and Health University of Sydney St Leonards NSW 2065 Australia; ^5^ Chair of Inorganic and Metal–Organic Chemistry Department of Chemistry Technical University of Munich Lichtenbergstrasse 4 85747 Garching Germany; ^6^ Department of Industrial Chemistry “Toso Montanari” Universita' degli Studi di Bologna Viale Risorgimento 40136 Bologna Italy; ^7^ Center for Chemical Catalysis - C3, Alma Mater Studiorum Università di Bologna Viale Risorgimento 4 40136 Bologna Italy; ^8^ Munich Data Science Institute (MDSI) Technical University of Munich Walther-von-Dyck Strasse 10 85748 Garching Germany

**Keywords:** Au nanoparticles, catalysis, N-heterocyclic carbenes, photothermal therapy, spectroscopy

## Abstract

N‐heterocyclic carbenes (NHCs) have become attractive ligands for functionalizing gold nanoparticle surfaces with applications ranging from catalysis to biomedicine. Despite their great potential, NHC stabilized gold colloids (NHC@AuNPs) are still scarcely explored and further efforts should be conducted to improve their design and functionalization. Here, the ‘bottom‐up’ synthesis of two water‐soluble gold nanoparticles (**AuNP‐1** and **AuNP‐2**) stabilized by hydrophilic mono‐ and bidentate NHC ligands is reported together with their characterization by various spectroscopic and analytical methods. The NPs showed key differences likely to be due to the selected NHC ligand systems. Transmission electron microscopy (TEM) images showed small *quasi*‐spherical and faceted NHC@AuNPs of similar particle size (ca. 2.3–2.6 nm) and narrow particle size distribution, but the colloids featured different ratios of Au(I)/Au(0) by X‐ray photoelectron spectroscopy (XPS). Furthermore, the NHC@AuNPs were supported on titania and fully characterized. The new NPs were studied for their catalytic activity towards the reduction of nitrophenol substrates, the reduction of resazurin and for their photothermal efficiency. Initial results on their application in photothermal therapy (PTT) were obtained in human cancer cells in vitro. The aforementioned reactions represent important model reactions towards wastewater remediation, bioorthogonal transformations and cancer treatment.

## Graphical abstract

**Small water‐soluble** N‐heterocyclic carbene‐stabilized gold nanoparticles (NHC@AuNPs) were synthesized via the bottom‐up approach and showed suitability for applications in catalysis and biomedicine.

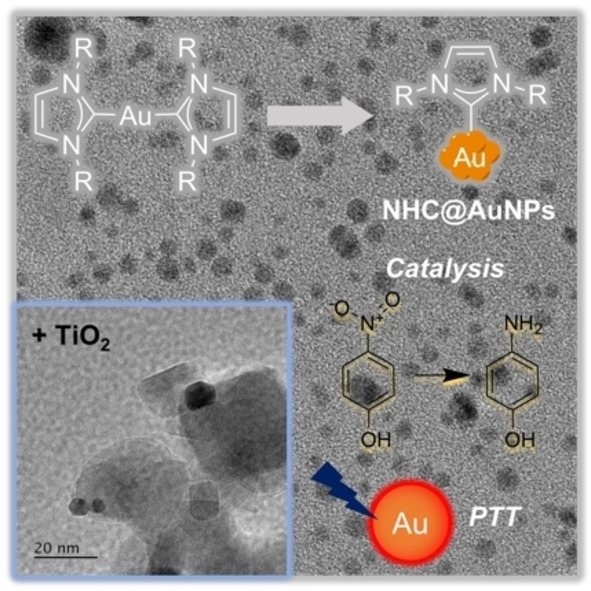

## Introduction

The remarkable properties of gold, including its high stability, unique spectral and magnetic properties and biocompatibility, made this noble metal a key material in nanotechnology. Gold nanoparticles (AuNPs) are amongst the most well‐studied metal‐based nanostructures, with applications in catalysis, sensing, drug delivery, bioimaging and photonics.^[1]^ Although, over the years, protocols for the synthesis of AuNPs with precise sizes and shapes have been refined,^[2]^ their surface chemistry remained almost unchanged for decades. Recently, N‐heterocyclic carbenes (NHCs) have emerged as ‘smart’ surface ligands for metal NPs due to their ability to form strong covalent bonds to metallic surfaces and to their versatility with respect to functionalization.^[3]^ In addition to the strong σ‐donor properties of NHC ligands, ligand surface adsorption and additional interactions of NHC wingtips seem to contribute to the overall stability of the NHC binding to Au(0) species.[^3a^, ^4^] In this context, two main approaches have been applied for the synthesis of AuNPs stabilized by NHC ligands (NHC@AuNPs): either by reducing Au(I) NHC complexes (the ‘bottom‐up’ approach) or by replacing labile ligands at the AuNP surface with NHCs (the ‘top‐down’ approach).

The aqueous stability of AuNPs is an important prerequisite for their biomedical applications, as well as to perform catalysis in aqueous environment, and is a great challenge in nanotechnology. In this context, NHCs can be exploited to confer ‘hydrophilic’ character to the nanomaterials as recently highlighted by a few literature reports.^[5]^ In 2015, following the ‘bottom‐up’ approach, the first example of water‐soluble NHC@AuNPs was reported by MacLeod and Johnson,^[5c]^ featuring PEGylated NHC ligands. More recently, Pleixats and co‐workers^[5g]^ reported the use of PEGylated imidazolium and tris‐imidazolium salts containing triazole linkers as stabilizers for the preparation of water‐soluble gold nanoparticles by reduction of tetrachloroauric acid with sodium borohydride (NaBH_4_) at room temperature.

In 2017, Crudden and co‐workers obtained water‐soluble NHC@AuNPs (2–4 nm size) by the ‘bottom‐up’ approach, reducing a bis‐NHC Au(I) complex featuring a carboxylated benzimidazolydene scaffold with NaBH_4_ in aqueous solution.^[5d]^ For the first time, the NHC@AuNPs were tested for biomedical applications as possible probes for photoacoustic imaging. In 2019, the groups of Chin and Reithofer achieved water‐soluble NHC@AuNPs by the ‘bottom‐up’ approach using NHC ligands derived from a *N*‐acetyl‐L‐histidine ethyl ester scaffold, featuring either a methyl or isopropyl group on the wingtips of the imidazole ring.^[5f]^ The obtained AuNPs (ca. 4 nm size) showed excellent stability in physiological conditions, including in the presence of the intracellular reducing agent glutathione (GSH).

In parallel, water‐soluble AuNPs stabilized by negatively charged NHC ligands, bearing either sulfonate or carboxylate groups as wingtip substituents, were synthesized following the ‘top‐down’ approach.^[5b]^ The AuNPs were formed using the didodecylsulfide (DDS)‐stabilized AuNPs as precursors in a biphasic mixture of hexane and dimethylformamide. The resulting NHC@AuNPs showed a mean size of 4.7±1.6 nm.^[5b]^ Also following the ‘top‐down’ approach, Camden, Jenkins and co‐workers reported a general method for the synthesis of NHC@AuNPs with protic groups and with an average diameter larger than 15 nm.^[5a]^ Moreover, Ravoo, Glorius and co‐workers^[5e]^ described water‐soluble bimetallic NHC@Au_x_Pd_y_NPs (ca. 4 nm) combining the advantageous properties of both Au and Pd nanoparticles. The latter were shown to be active as biomimetic catalysts for the aerobic oxidation of D‐glucose into gluconic acid and hydrogen peroxide. The NPs also showed sensitivity to the diastereomers of D‐glucose (D‐mannose and D‐galactose), whereas no oxidation was observed for D‐fructose.^[5e]^

Interestingly, the concept of bidentate NHCs on gold nanoparticles was first described by Crudden and co‐workers^[6]^ for the preparation of highly stable nanomaterials. Later on, Johnson and co‐workers^[7]^ used bidentate thiolate‐NHC−Au(I) moieties to tune the size and shape of the NPs to obtain nanorods. In this case, the applied ‘top‐down’ synthetic strategy was inspired by the tendency of NHC ligands to abstract a gold atom from the surface lattice to generate translationally mobile NHC@Au adatom complexes.^[8]^ The resulting thiolate‐NHC‐stabilized Au nanorods were water‐soluble benefiting from the functionalization of the NHC backbone with triazole‐conjugated polyethylene glycol, and could be applied for photothermal therapy (PTT) in vitro. Gold nanoparticle‐based PTT has recently been extensively studied in cancer treatment, due to the local hyperthermia induced by their plasmonic photothermal effect on cancer cells or tissues.^[9]^

Overall, these examples show that the particle size, particle size distribution, shape and stability of NHC‐coated AuNPs strongly depend on the structure of the NHC ligands and the reaction conditions used for their synthesis.

**Scheme 1:**
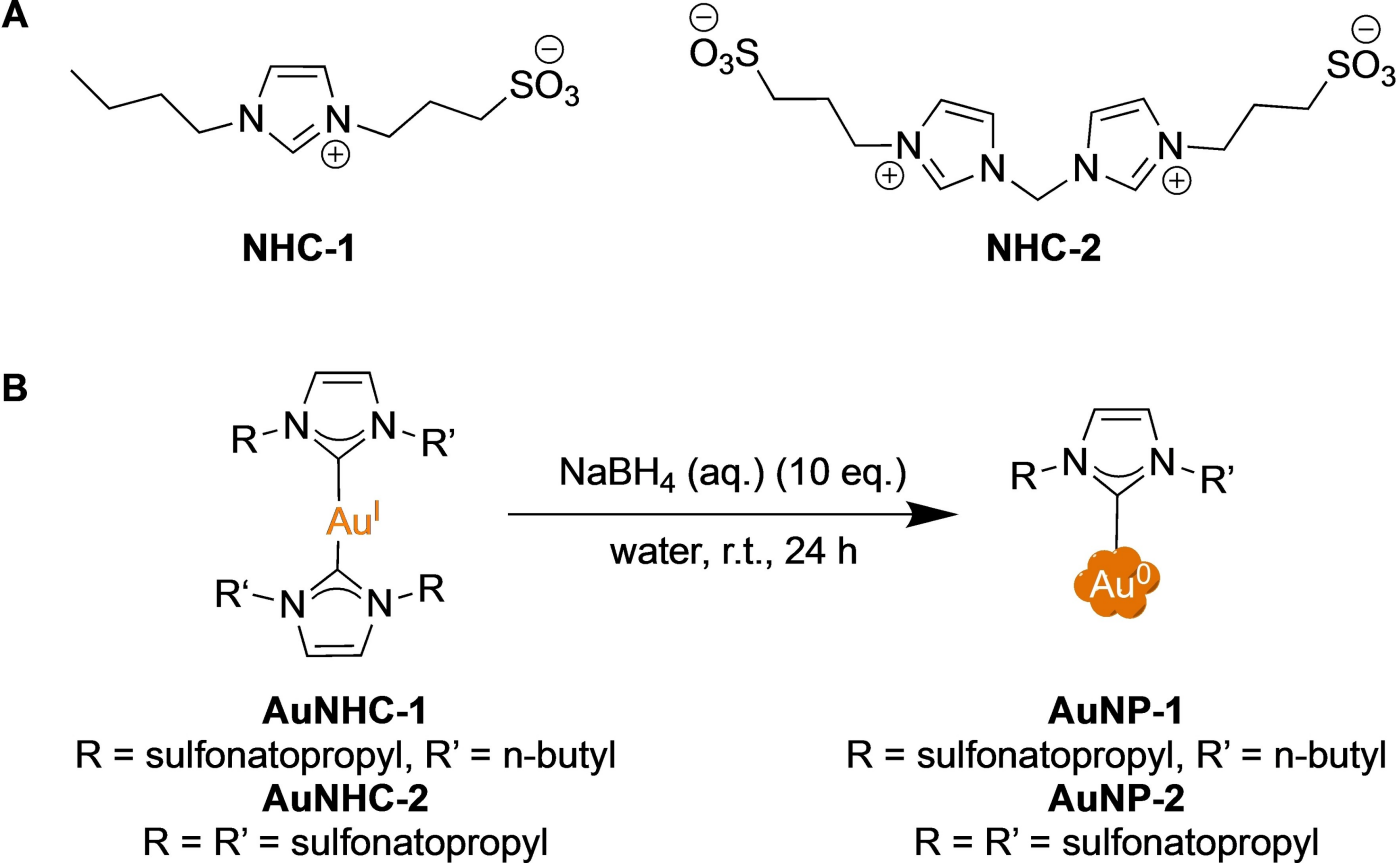
**A**) Structures of ligands (**NHC‐1** and **NHC‐2**) used in this work. **B**) Synthesis of water‐soluble NHC@AuNPs (**AuNP‐1** and **AuNP‐2**) by direct reduction of bis‐NHC Au(I) complexes (**AuNHC‐1** and **AuNHC‐2**) with NaBH_4_ in MilliQ water.

Despite these encouraging results, the number of NHC@AuNPs is still limited and numerous applications remain unexplored, particularly in the area of biomedicine. Therefore, taking advantage of our experience in designing hydrophilic metal NHC complexes for biological applications,^[10]^ we selected mono‐ and bidentate NHC ligands featuring wing‐tip anchored sulfonate groups to achieve two bis‐NHC Au(I) complexes (Scheme 1).[^10^, ^11^] The latter were then used to synthesize NHC@AuNPs via the ‘bottom‐up’ approach. It should be noted that similar NHC ligands were previously reported to stabilize Au, Pd and Pt nanoparticles with sizes ranging between 1.6 to 4.7 nm depending on the metal (e. g. 4.7±1.5 nm in the case of AuNPs).[^5b^, ^12^]

The herein reported NPs were then characterized by a plethora of spectroscopic and analytical methods, including UV‐Visible (UV‐Vis) absorption spectroscopy, ^1^H NMR spectroscopy, inductively coupled plasma mass spectrometry (ICP‐MS), Fourier‐transform infrared attenuated total reflectance (FTIR‐ATR) spectroscopy, transmission electron microscopy (TEM), thermogravimetric analysis (TGA) and X‐ray photoelectron spectroscopy (XPS). The catalytic performance of the synthesized NHC@AuNPs, unsupported and supported on titania, was evaluated in the catalytic reduction of 4‐nitrophenol, and similar substrates, as a model reaction for wastewater remediation, as well as to reveal structure‐activity relationships. The unsupported NHC@AuNPs were also evaluated for the reduction of resazurin by NH_2_OH in water, leading to the formation of a fluorescent product. This reaction was chosen as a model for gold‐mediated bioorthogonal transformations by the NPs in aqueous environment.^[13]^ Moreover, in preliminary studies, we also investigated the possible application of the NPs for PTT in human prostate adenocarcinoma PC‐3 cells in vitro, as an important emerging application for cancer treatment.

## Results and Discussion

### Synthesis and Characterization of NHC@AuNPs

Initially, sulfonated ligands **NHC‐1** and **NHC‐2** (Scheme 1A) were synthesized according to previously reported procedures.[^10^, ^14^] Afterwards, the two water‐soluble Au(I) NHC complexes (**AuNHC‐1** and **AuNHC‐2**) were formed via transmetalation from their respective Ag(I) bis‐NHC complexes in the presence of the Au(I) precursor [Au(SMe_2_)Cl] (SMe_2_=dimethylsulfide).[^10^, ^11^] The water‐soluble NHC@AuNPs were then obtained by the ‘bottom‐up’ approach involving the direct reduction of the Au(I) NHCs with sodium borohydride (NaBH_4_, 10 equiv) in MilliQ water (Scheme 1B).

To facilitate full reduction, the AuNP solutions were stirred at room temperature for 24 h. To further remove possible unreacted species on the surface of the AuNPs, the solutions were subjected to dialysis against water for ca. 48 h (see Experimental for details). The NHC@AuNPs were obtained in moderate yields: 18–31 % based on metal content determined by ICP‐MS after purification. AuNP formation was assessed by UV‐Vis spectroscopy in unbuffered MilliQ water confirming the presence of the typical surface plasmon resonance (SPR) band between 510–520 nm for metallic AuNPs with mean Au particle size around 2–5 nm.^[15]^

Representative absorption spectra of **AuNP‐2** in comparison to the colourless solution of the Au(I) NHC precursor are shown in Figure S0 in the Supporting Information, showing the appearance of the classical SPR band after 24 h from addition of NaBH_4_ in water.

**Figure 1:**
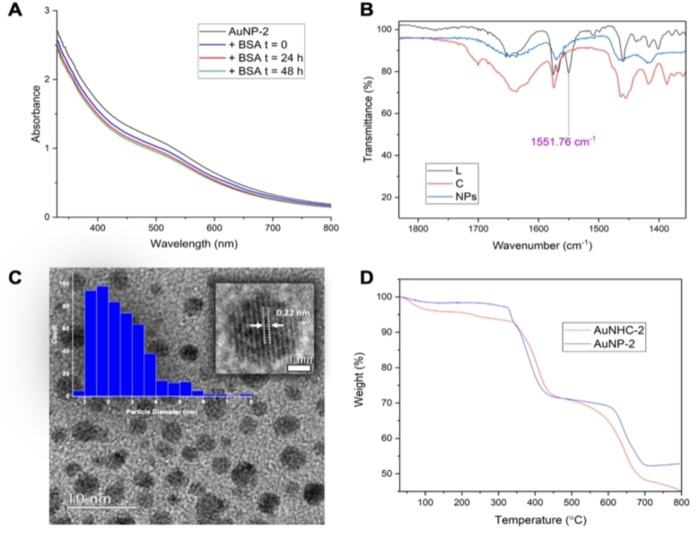
**A**) UV‐Vis absorption spectra of **AuNP‐2** in PBS 1x (pH 7.4) (black trace)+BSA (17 mM) at time 0 (blue trace), after 24 h and 48 h (red and green traces), respectively. **B**) FTIR‐ATR spectra of ligand **NHC‐2** (L, black trace), complex **AuNHC‐2** (C, red trace) and **AuNP‐2** (NHC@AuNPs, blue trace); imidazolium C−H ring stretch signal (1551.76 cm^−1^) only seen in the free imidazolium ligand. **C**) Particle size histogram of **AuNP‐2** displaying an average size of 2.6±1.2 nm with representative TEM image (scale bar: 10 nm) and insert: HR‐TEM of individual particle with 0.22 nm lattice spacing. **D**) Overlap of TG curves for **AuNHC‐2** (red trace) and **AuNP‐2** (blue trace).

Stability in physiological conditions is paramount for the use of AuNPs in biomedical applications; therefore, the stability of **AuNP‐1** and **AuNP‐2** was assessed in both MilliQ water and PBS 1x (phosphate buffered solution, pH 7.4) over 15 h by absorption spectroscopy. In these conditions, both colloidal systems show similar high stability (Figures S1–S2 in the Supporting Information). The biological compatibility of the NPs was then investigated further by addition of the intracellular reducing agent glutathione (GSH, 2 mM, Figure S3) as well as in the presence of bovine serum albumin (BSA, Figure 1A reports representative spectra for **AuNP‐2**). Both AuNPs showed high stability in these conditions with minor variations in the UV‐Vis absorption spectra as a function of time (0–24 or 48 h), as seen in Figures 1A and S3 in the SI.

**Figure 2:**
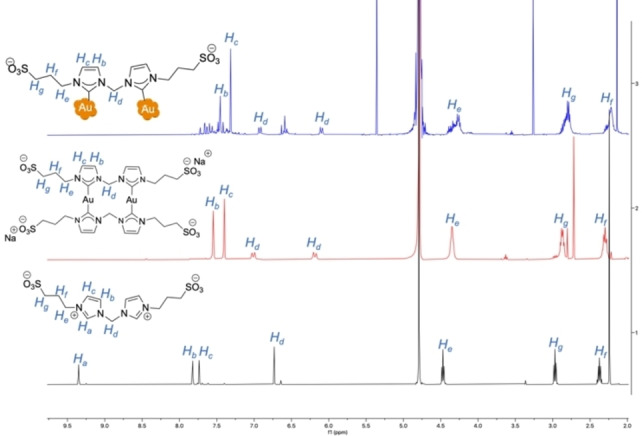
^1^H NMR spectra of imidazolium ligand **NHC‐2** (bottom, black), complex **AuNHC‐2** (middle, red) and **AuNP‐2** (top, blue) in D_2_O.

The NHC@AuNPs were further characterized using ^1^H NMR spectroscopy to gain insight into the ligand structure retained on the NP surface, and to confirm the loss of the imidazolium proton due to the NHC binding to the NP surface (Figures 2 and S4). Representative ^1^H NMR spectra of **AuNP‐2** with its Au(I) precursor (**AuNHC‐2**) and imidazolium ligand (**NHC‐2**) in D_2_O are shown in Figure 2. As expected, the imidazolium proton (H_a_ at 9.35 ppm) was only observable in the spectrum of the **NHC‐2** ligand. The absence of the imidazolium proton in the NHC@AuNPs was further confirmed using FTIR‐ATR spectroscopy. The results obtained for **AuNP‐2** are reported in Figure 1B and show the absence of the imidazolium peak at 1551.8 cm^−1^ in the spectra of both **AuNP‐2** and **AuNHC‐2**, in line with literature values.[^5c^, ^16^] This trend was also observed for **AuNP‐1** (Figure S5).

TEM analysis was also carried out on the synthesized NPs to evaluate their average particle size and size particle distribution, as well as the shape of the NHC@AuNPs. The NP films were formed by drop‐casting their solution in acetone onto the carbon grid and leaving to dry before images were acquired. The TEM images showed small *quasi*‐spherical and faceted NHC@AuNPs of similar particle size and narrow particle size distribution: **AuNP‐1** (2.3±1.1 nm, Figure S6) and **AuNP‐2** (2.6±1.2 nm, Figures 1C and S7), respectively. It is evident from these results that the synthesis of small NHC@AuNPs with narrow particle size distribution was successful and comparable to alternative experimental colloidal methods; for example, the Brust method and colloidal methods using PVA (polyvinyl alcohol) and PVP (polyvinyl pyrrolidone) as stabilizers.^[17]^

The extent of NHC functionalization on the NP surface was then assessed using TGA and performed on both the NHC@AuNPs and their corresponding Au(I) NHC complexes. The measurements were recorded in a N_2_ environment (20 mL/min) and the samples heated with a ramp rate of 5 °C min^−1^ from 30 to 800 °C. The estimated percentage ratio of Au:NHC in each case is reported in Table 1. The obtained results showed that, in the case of **AuNP‐1**, up to 800 °C the TG curve displays minimal weight loss as a percentage with respect to the Au(I) complex **AuNHC‐1**, which can be associated with the percentage of organic component present on the NP surface (Figure S8). Accordingly, the estimated percentage of ligand functionalization at the **AuNP‐1** surface is lower than for its corresponding Au(I) precursor (7 vs. 61 %, respectively), indicating loss of ligand upon NP formation. Conversely, the TG curves for **AuNP‐2** and its corresponding **AuNHC‐2** appeared almost identical highlighting the retainment of the ligands during NP formation possibly giving a larger surface coverage (Figure 1D). The ratio of Au:NHC (% weight ratio) was determined by TGA data following the method applied by Johnson et al.^[5c]^ (Table 1). In the case of **AuNP‐2**, the obtained result of Au:NHC % of 53 : 47 is in line with the ratio reported for other NHC@AuNPs systems,^[18]^ evidencing that ligand retention on the NP surface is commonly observed at least for colloids obtained via the ‘bottom‐up’ approach.

**Table 1:** Structural properties of the investigated NHC@AuNPs.

NHC@AuNP	Au particle size^[a]^ [nm]	Au:NHC^[b]^ [%]	Au content [%]	Au(0) content^[d]^ [%]
AuNP‐1	2.3±1.1	93 : 7	12.6	72
AuNP‐2	2.6±1.2	53 : 47	29	38

[a] Average diameter of the gold nanoparticles and standard deviation values as determined by TEM analysis. [b] NP metal and ligand ratio percentage as determined by TG analysis. [c] Au content percentage determined by ICP‐MS. [d] Au(0) content percentage estimated by XPS analysis.

**Figure 3:**
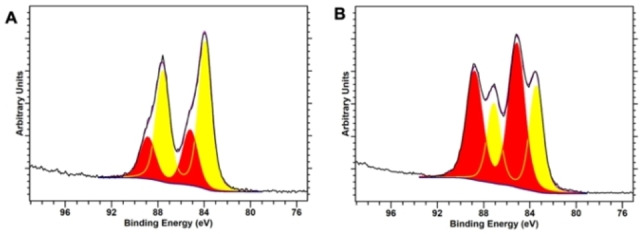
XPS spectra of the Au 4 f region for the NHC@AuNPs (Au(I) in red and Au(0) in yellow) of **A**) **AuNP‐1** and **B**) **AuNP‐2**.

XPS analysis was then performed on the NHC@AuNPs to gain information on the surface atoms and their atomic surface concentration, as well as the oxidation states of the possible Au species present on the NP surface (Figures S9 and S10, as well as Table S1 in the Supporting Information). Evidence for the presence of the NHC ligand on the AuNP surface has already been assessed by this method in the literature due to the indicative C 1s and N 1s signals commonly observed.[^6^, ^18^] Beginning with the C 1s peaks, both AuNPs showed three characteristic peaks at ca. 285, 286 and 288/289 eV. The peaks at 285 and 286 eV can be confidently assigned to the C−C and C−N bonds of the corresponding ligands, respectively, in line with literature values.[^18b^, ^19^] The high binding energy peak at 288/289 eV can be attributed to a highly oxidized carbon species such as a carbonate which could have been introduced during mounting of the sample onto the glass slide for analysis.^[20]^ In addition, both NHC@AuNPs feature a peak around 401 eV corresponding to the N 1s peak present in the NHC structure.[^6^, ^18^, ^19^] Finally, the XPS data of the Au 4 f region of both NPs showed the presence of two oxidation states, characterized by Au 4f_7/2_ peak binding energies of 83 and 85 eV, corresponding to Au(0) and Au(I) respectively (Figure 3 and Table S1). Based on the peaks’ areas, the percentage concentration of Au(I) in **AuNP‐2** (62 %) is significantly larger than in **AuNP‐1** (28 %). Overall, this data led us to postulate that NHC@AuNPs exhibit a monolayer of molecular Au(I) species, presumably as Au(I) NHC complexes, surrounding the Au(0) core, in accordance with previous studies.[^4^, ^18b^, ^21^]

However, we cannot exclude the presence of segregated Au(0) and Au(I) species. The exact nature of these Au(I) species still remains unsolved and further investigation is needed using advanced characterization, such as X‐ray absorption fine structure (XAFS) spectroscopy, high‐angle annular dark‐field (HAADF) imaging and high‐resolution (HR) TEM.[^18b^, ^21^]

### Catalytic Activity of NHC@AuNPs

**Figure 4:**
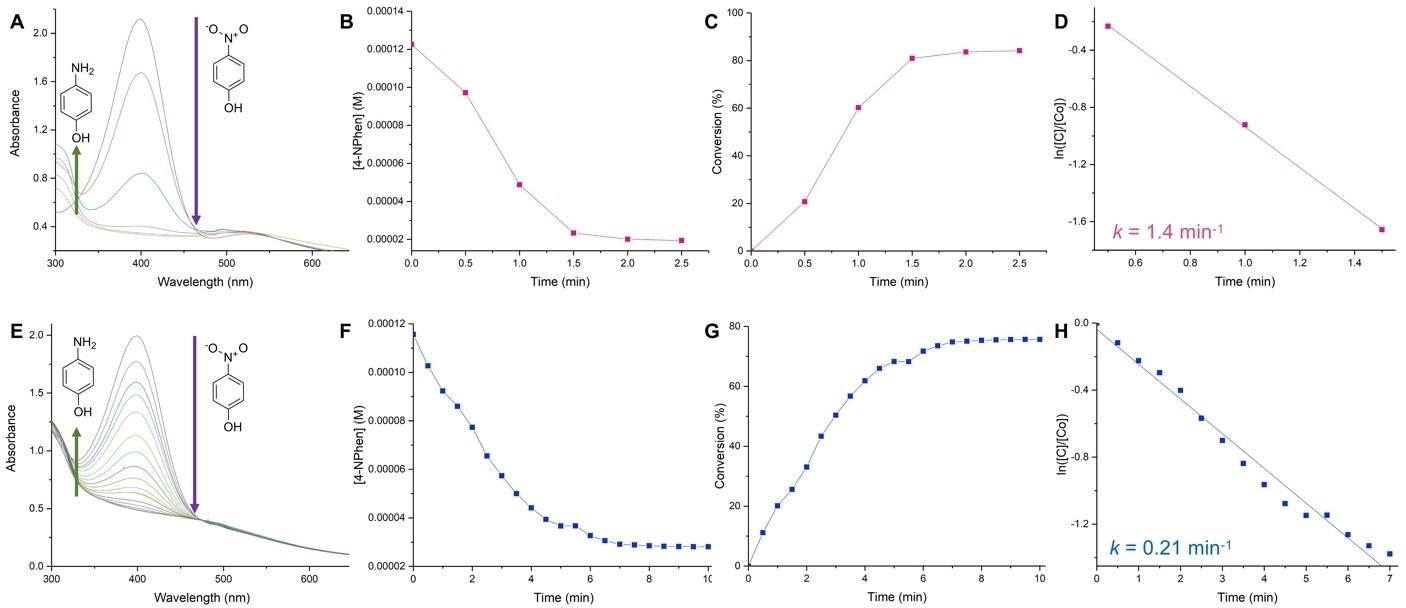
UV‐Vis absorption kinetic studies for the reduction of 4‐nitrophenol (4‐NPhen) catalyzed by NHC@AuNPs in water at r.t. **A**) **AuNP‐1** (0.2 mg) over 2.5 min, with a spectrum recorded every 30 sec, **E**) **AuNP‐2** (0.3 mg) over 10 min, with a spectrum recorded every 30 sec. Plots of [4‐NPhen] vs. time monitored at 400 nm for **B**) **AuNP‐1** and **F**) **AuNP‐2**, respectively. Plots of % substrate conversion vs. time for **C**) **AuNP‐1** and **G**) **AuNP‐2**, respectively. Plots of ln([C]/[C_o_]) vs. time for the reduction of 4‐nitrophenol showing first order kinetics, and calculated rate constants for **D**) **AuNP‐1** and **H**) **AuNP‐2**, respectively.

Following full characterization of the NHC@AuNPs, their catalytic performance was then assessed using the model 4‐nitrophenol reduction reaction. The addition of excess NaBH_4_ allows the reaction to be considered as *pseudo*‐first order with respect to the concentration of 4‐nitrophenol (4‐NPhen). The formation of the reduced 4‐aminophenol species in the presence of AuNPs can be monitored at 300 nm by UV‐Vis spectroscopy over time, in concomitance to the disappearance of the corresponding 4‐nitrophenol band at 400 nm.^[22]^ A calibration plot was first measured by plotting the absorbance at 400 nm of different concentrations of 4‐nitrophenol (Figure S11) to then allow us to determine the rate constant. The UV‐Vis kinetic studies for both **AuNP‐1** and **AuNP‐2** are shown in Figure 4. **AuNP‐1** shows high catalytic efficiency with substrate conversion reaching 81 % already after 1.5 min (Figure 4A−C). It should also be noted that an induction period is present for **AuNP‐1** from 0 to 0.5 min whereby the reaction rate appears to be slower (Figure 4B−C), indicating possible restructuring of the active sites of the catalyst. The UV‐Vis kinetic study of **AuNP‐2** (Figure 4E−G) shows that the reaction requires longer time (>6 min) to reach a plateau until ca. 80 % substrate conversion. Specifically, after 1 min, substrate conversion reaches 60 % and 20 % in the case of **AuNP‐1** and **AuNP‐2**, respectively.

The rate constant for each NHC@AuNP was also calculated by plotting the natural logarithm for the change in substrate concentration over reaction time. In the case of **AuNP‐1**, due to the observed induction period, the apparent rate constant was calculated to be between 0.5 to 1.5 min resulting in a value of *k*=1.4 min^−1^ (Figure 4D).

In the case of **AuNP‐2**, a rate constant of 0.21 min^−1^ was calculated (Figure 4H). ICP‐MS analysis was also performed on the NHC@AuNP samples to determine the mass of ^197^Au in each case. The obtained results showed a higher overall gold content for **AuNP‐2** (29 % per mg) vs. **AuNP‐1** (12.6 % per mg). Despite this result, the lower catalytic activity of **AuNP‐2** can be explained considering the lower % of Au(0) in its samples, compared to **AuNP‐1**, as assessed by XPS (Table 1). Moreover, the observed high ligand‐surface coverage in **AuNP‐2** could also contribute to the reduction of catalytic performance due to the coverage of the accessible Au sites by the NHC ligand (Table 1). In order to provide evidence that the catalyst activity is not due to Au(I) species present in **AuNP‐2**, we have attempted the 4‐NP reduction using the Au(I) complex **AuNHC‐2** in presence of excess NaBH_4_. As expected, the substrate conversion is irrelevant even after a few hours (Figure S12). This experiment further demonstrates the colloidal nature of the catalytically active gold species in **AuNP‐2**.

Additionally, the versatility of the AuNP catalysts was explored by investigating the reduction rate of 2‐nitrophenol and 3‐nitrophenol substrates (calibration plots can be found in Figures S13 and S14). In both cases the conversions to their respective aminophenol species was significantly lower (ca. 2‐ or 4‐fold decrease, respectively) compared to 4‐nitrophenol; however, both catalysts showed relatively similar rate constants (Figures S15–S18).

**Figure 5:**
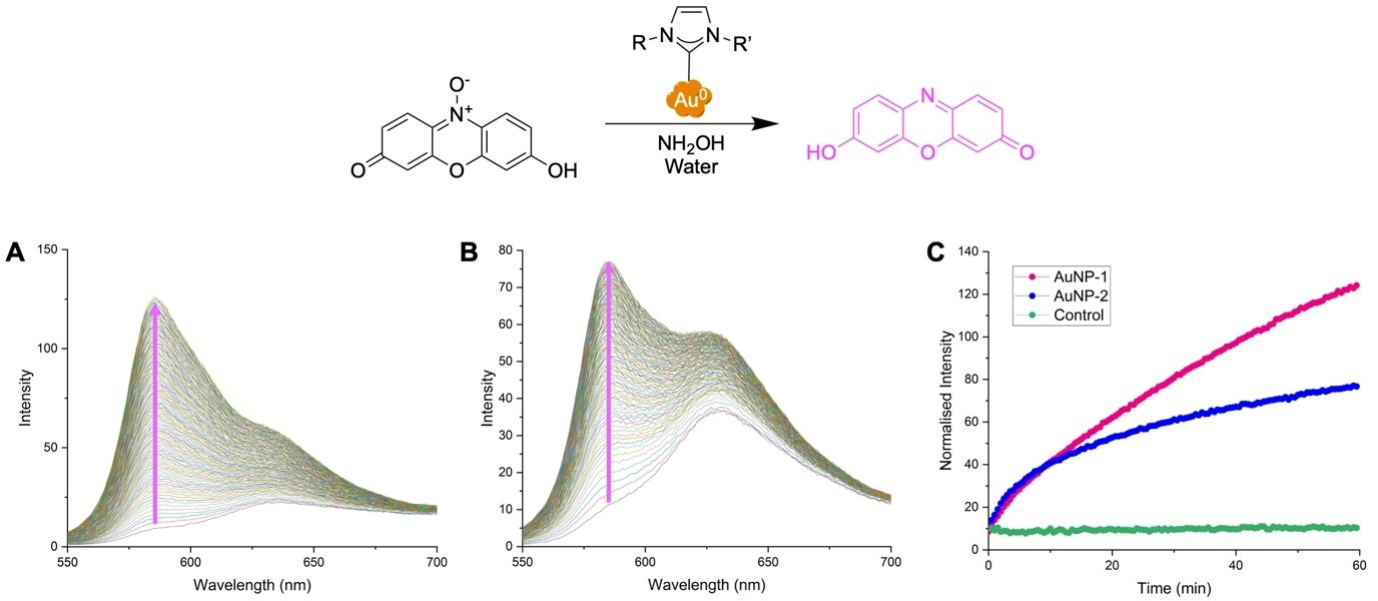
Fluorescence emission studies for the reduction of resazurin catalyzed by NHC@AuNPs in water at r.t. with NH_2_OH. Spectra were recorded every 30 sec over 1 h. **A**) **AuNP‐1** (1 mg) and **B**) **AuNP‐2** (1 mg), respectively. Magenta arrows highlight the increase in intensity of the resorufin product at 584 nm. **C**) Comparison of the fluorescence emission intensity of the resorufin product at 584 nm over time, for reactions catalyzed by NHC@AuNPs vs. control (without NPs).

In order to assess the catalytic efficiency of the NPs towards other biologically relevant reactions, we studied the NHC@AuNPs activity towards the reduction of resazurin by NH_2_OH in water, and followed the formation of the fluorescent product resorufin as a function of time (Figure 5). The obtained results show that both NP systems are catalytically active, and follow a similar trend as observed for the 4‐NPhen reaction, with **AuNP‐1** being more active than **AuNP‐2**.

### NHC@AuNPs Supported on Titania

Supports are often utilized in the synthesis of colloidal AuNPs to provide increased stability, as well as potentially improving their catalytic performance with increased selectivity, both directly or indirectly.^[23]^ However, the use of a support in combination with surface stabilizing ligands requires careful optimization as both features can decrease the surface accessibility for catalytic reactions.^[3a]^ Examples of supported NHC@AuNPs have been recently reviewed by Reithofer and co‐workers;^[3a]^ however, the area is still very much in its infancy and only a few examples of metal NPs supported on carbon, silica or graphene oxide have been reported. Therefore, the NHC@AuNPs were supported on titania (TiO_2_) as a common reference support used for catalytic reactions, using an adapted colloidal method.^[24]^ In detail, the pre‐formed NPs were dissolved in MilliQ water before addition of the titania support, the sample was then left under vigorous stirring overnight at room temperature to facilitate the immobilization of the NPs.

Of note, the white titania became lilac in colour after a few minutes indicating AuNP immobilization. XPS and TEM were used to examine if the NHC@AuNPs had been successfully immobilized and to observe whether they had retained a similar particle size and morphology. XPS data indicates that **AuNP‐1/TiO_2_** contain all Au(0), whereas **AuNP‐2/TiO_2_** also contain a percentage of Au(I) (Figure S19 and S20 and Table S2 in the Supporting Information).

In both samples the concentration of NHC@AuNPs appears to be low compared to the theoretical loading as confirmed by ICP‐MS, with 47 % of Au in the supported **AuNP‐1/TiO_2_**, and even lower (only 7 %) in the case of **AuNP‐2/TiO_2_**.

**Figure 6:**
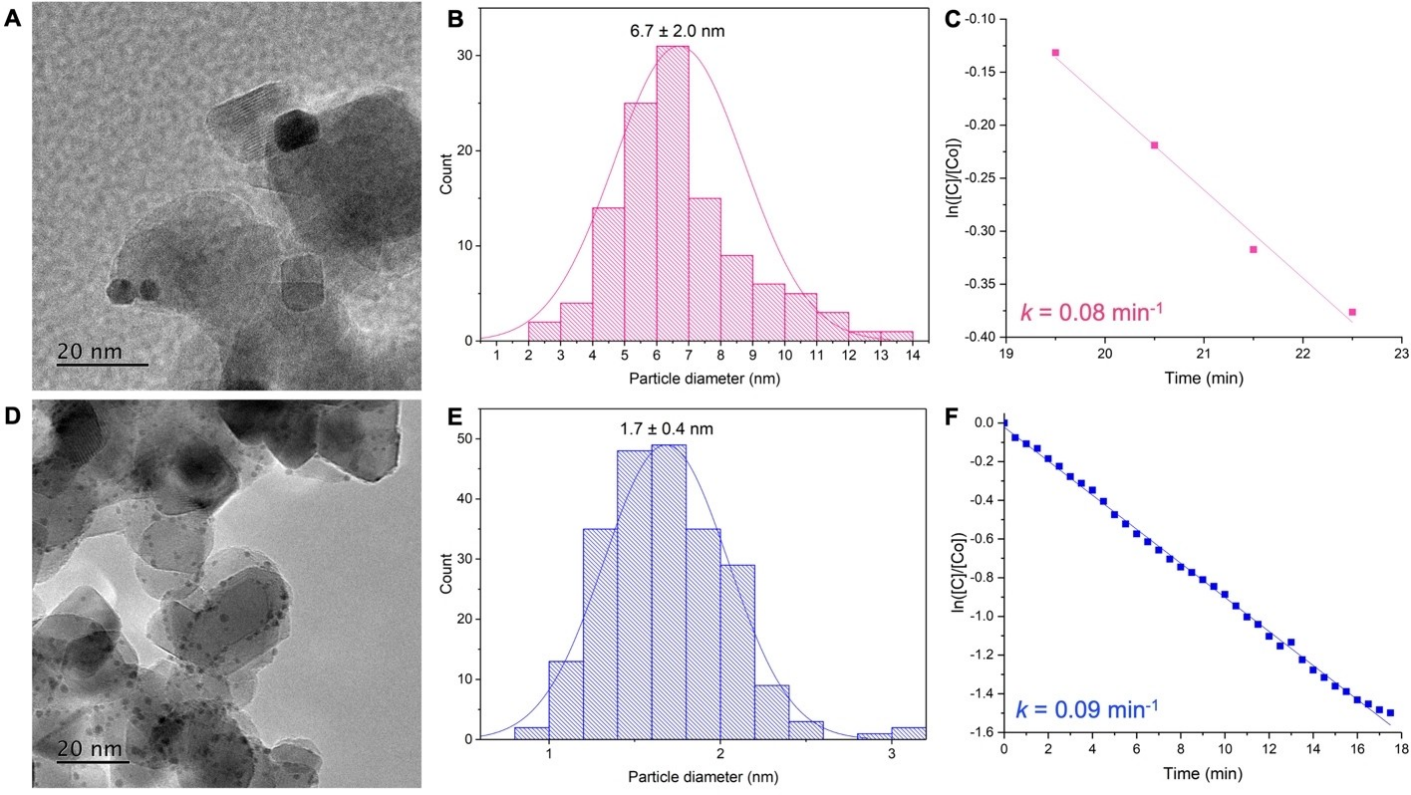
TEM images of **A**) **AuNP‐1/TiO_2_** and **D**) **AuNP‐2/TiO_2_** with scale bars, along with their corresponding particle size histograms **B**) 6.7±2.0 nm and **E**) 1.7±0.4 nm, respectively. Plot of ln([C]/[C_o_]) vs. time for the reduction of 4‐nitrophenol with **C**) **AuNP‐1/TiO_2_** and **F**) **AuNP‐2/TiO_2_** showing first order kinetics and calculated rate constants.

Interestingly, the TEM images of **AuNP‐1/TiO_2_** (Figures 6A and S21) and particle size distribution (Figure 6B) show the presence of larger NHC@AuNPs (6.7±2.0 nm) compared to the unsupported NPs (2.3±1.1 nm) and they do not appear to be well dispersed. Conversely, the TEM images of **AuNP‐2/TiO_2_** (Figures 6D and S21) shows the presence of smaller NHC@AuNPs with a narrower particle size distribution (1.7±0.4 nm, Figure 6E) compared to **AuNP‐1/TiO_2_** and unsupported **AuNP‐2** (2.6±1.2 nm). Moreover, the binding energy of the Au(0) in **AuNP‐1/TiO_2_** is lower than its unsupported precursor (82.9 vs. 83.9 eV, Table S1 and S2) which has typically been attributed to increased particle size of the NPs deposited on the TiO_2_ or increased charge transfer between Au(0) and TiO_2_.^[25]^ In this case both phenomena may occur, with the increased charge transfer possibly contributing to the higher loading of **AuNP‐1** on TiO_2_ compared to **AuNP‐2** (47 % vs. 7 %, respectively); the latter not featuring a lower binding energy of the Au(0). Furthermore, the reduced charge transfer for **AuNP‐2/TiO_2_** could explain the NPs smaller size compared to the unsupported ones; as the larger, heavier particles of **AuNP‐2** would find it harder to migrate through the solution to undergo interaction, adsorption and adhesion to the support.

### Catalytic Activity of NHC@AuNPs/TiO_2_

Preliminary tests were performed to assess whether the supported NHC@AuNPs had retained their catalytic performance for the 4‐nitrophenol reduction reaction upon immobilization. Although a direct comparison cannot be made with the unsupported AuNPs due to different concentrations of gold and ratios to substrates, the supported NPs do appear to retain at least some of their catalytic performance; although **AuNP‐1/TiO_2_** shows a much greater induction period compared to the respective unsupported NPs and a considerable decrease in the rate constant *k*=0.08 min^−1^ (Figure 6C and S22 in the Supporting Information). However, this can be expected due to the larger size of the supported NPs and the presence of the TiO_2_ which could result in reduced access to the NP active sites. Loading of **AuNP‐2** on TiO_2_ appears to have less of an effect on the calculated rate constant *k*=0.09 min^−1^ (Figure 6F and S23 in the Supporting Information) which can be attributed to the preservation of the small NP size.

The recyclability of the supported NHC@AuNPs was then assessed using the same conditions as above, with subsequent removal of the filtrate and washing of the NPs with water. The filtrate and wash after each cycle were then analyzed by ICP‐MS and the NHC@AuNPs were left to dry overnight before the addition of fresh 4‐nitrophenol and NaBH_4_ solutions. In total, three cycles of the 4‐nitrophenol reduction were performed. In the case of **AuNP‐1/TiO_2_**, an overall decrease in the rate of substrate conversion vs. time after the first cycle was recorded (Figures S24 and S25 in the Supporting Information); however, a high % of substrate conversion is still observed after each cycle. In order to determine whether leaching of Au from the supported NPs is responsible for the decrease in catalytic efficiency, ICP‐MS was performed on the filtrate and wash fractions after each cycle. The results showed that overall only ca. 0.4 % of the initial amount of Au was present in the filtrated solution. This was also evidenced by the XPS analysis performed on the NHC@AuNPs before and after the three cycles, showing a similar % of the initial Au 4 f signal (Table S3). These results evidence that the colloidal Au nanoparticles were stable on the support, implying a strong metal‐support interaction. The observed deactivation could be due to either strong poisoning of the product on the active sites or surface restructuring of the active sites. XPS analysis showed a decrease of the carbon content after the reaction, and similar values of Au content before and after reaction; therefore, it is likely that chemical poisoning could be the main reason for the decreased substrate conversion observed.

**Figure 7:**
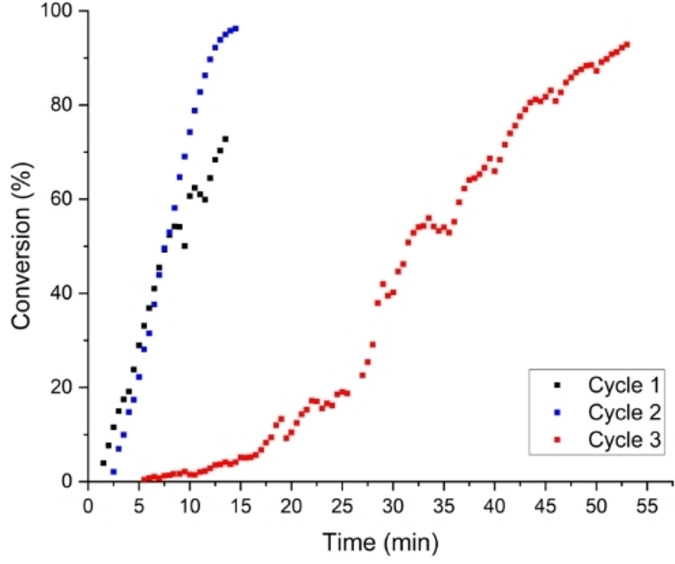
Plot of % substrate conversion vs. time for the reduction of 4‐nitrophenol with **AuNP‐2/TiO_2_** over three cycles.

Concerning **AuNP‐2/TiO_2_**, this catalytic system showed a high % of substrate conversion vs. time throughout the cycles (Figures 7, S26 and S27), with the substrate completely converted over ca. 15 min in the first two cycles. This high catalytic activity is maintained despite the significant leaching (ca. 99 %) observed in the filtrate and wash fractions after the first cycle by ICP‐MS. These results could be explained to a certain extent by (*i*) the removal of surface‐bound organic species after the first 4‐nitrophenol reduction step resulting in more available active sites for the subsequent cycle, (ii) the removal of the spectator species and as a consequence the restructuring of the active sites that can contribute to the observed high activity, as it has been shown previously by Flytzani‐Stephanopoulos et al.^[26]^ using leaching techniques to synthesise highly active gold catalysts, with uniform dispersion of gold atoms and nanoclusters. After cycle 2, minimal further leaching was observed, and no additional leaching was detectable after cycle 3. The dramatic Au loss across the cycles is also reflected in the XPS analysis as after cycle 3 the peaks of Au 4 f can no longer be identified (Table S4).

Moreover, one possible hypothesis could be that during the leaching the “spectator” sites have been removed and the presence of well‐dispersed and small size nanoparticles have remained on the surface of the catalyst. This hypothesis is based on recent work published by Hardacre and co‐workers^[27]^ who showed an effective chemical treatment for the re‐dispersion of AuNPs forming very small nanoparticles below 1.5 nm.

Furthermore, Flytzani‐Stephanopoulos and co‐workers^[28]^ have shown another effective chemical treatment for the selective removal of specific Au sites as spectators from the surface of the catalyst. In both cases the prepared nanomaterials showed higher catalytic activity. Unfortunately, due to the low amount of the catalyst, it was not possible to perform HRTEM analysis to verify this hypothesis, but only XPS analysis. In future studies we will focus on understanding better the restructuring of the active sites on the surface of the catalyst during chemical treatment and catalytic reactions.

### Photothermal Efficiency and PTT applications

**Figure 8:**
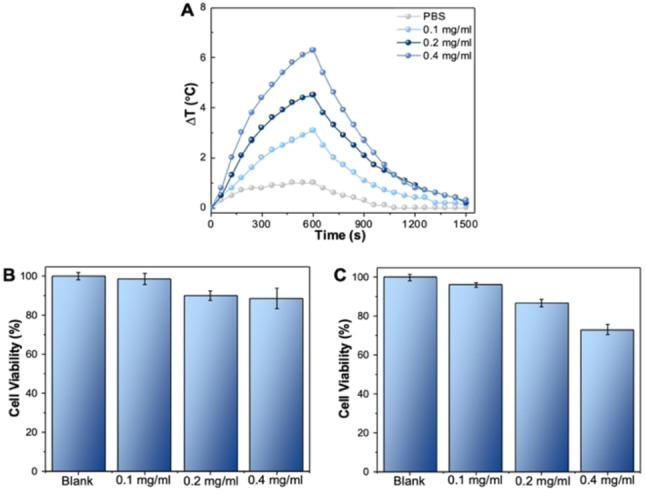
**A**) Photothermal effects of **AuNP‐2** at different concentration in 1x PBS buffer (pH 7.4) under laser irradiation, where the laser was first irradiated for 600 s and then removed. **B**) Effect on PC‐3 cell viability following **AuNP‐2** treatment at different concentrations for 24 h. **C**) Effect on PC‐3 cell viability following **AuNP‐2** treatment at different concentrations for 6 h followed by a 10 min laser irradiation (808 nm, 340 mW/cm^2^) and another 18 h incubation. Blank=1x PBS buffer (pH 7.4).

In a second phase of our study, we conducted preliminary investigations on the potential of water‐soluble NHC@AuNPs for applications in photothermal therapy of cancer. Prior to the PTT experiments, the photothermal efficiency of the **AuNP‐2** solution was evaluated with a near‐infrared (808 nm) laser source using a fluence of 340 Wcm^−2^. Detected by thermocouple, a time and concentration‐dependent temperature evaluation was observed for the **AuNP‐2** solution (Figure 8A). Since the **AuNP‐2** cannot resonate with the NIR irradiation directly, as they feature an SPR band at ca. 520 nm, the photothermal effect could be due to the two‐photon absorption and second harmonic generation.^[29]^ Consequently, the photothermal efficiency was calculated using Equations (1)–(3) (see Experimental) and was estimated to be ca. 25 % for 0.4 mg/mL **AuNP‐2** PBS solution under the NIR laser irradiation (Table S5). Whilst it is difficult to directly compare the data to existing literature on different types of AuNPs, particularly those with larger sizes commonly studied,^[30]^ this data is encouraging and shows that even small AuNPs can be exploited for PTT. Progressing to the biotoxicity and PTT evaluation, cell viability studies were performed in human PC‐3 prostate cancer cells in vitro. In absence of the NIR light irradiation, **AuNP‐2** showed acceptable biocompatibility with high PC‐3 cell viability in a range of concentrations (Figure 8B).

At the highest tested concentration (0.4 mg/mL) of **AuNP‐2**, cell viability was maintained (>88 %). However, the combination of **AuNP‐2** (0.4 mg/mL) and 10 min irradiation under the NIR source resulted in a notable decrease in cell viability due to local overheating (Figure 8C). Instead, no significant decrease of cell proliferation could be observed in PC‐3 cells incubated with 0.1 and 0.2 mg/mL **AuNP‐2**, respectively. These results are in good agreement with the concentration‐dependent photothermal performance observed in (Figure 8C). This can be attributed to the fact that the SPR effect is only strong enough to induce the local hyperthermia when there is an adequate amount of **AuNP‐2** to convert the energy of the laser into heat.^[31]^ It should be noted that, based on the previously mentioned ICP‐MS data, the Au content in **AuNP‐2** is only ca. 29 % per mg, which may result in reduced photothermal efficiency. Despite the need for further optimization, the observed PTT capability of **AuNP‐2** suggests that the obtained NHC@AuNPs are promising for cancer treatment upon laser irradiation.

## Conclusion

In conclusion, we have successfully synthesized two types of water‐soluble NHC@AuNPs (**AuNP‐1** and **AuNP‐2**) with narrow particle size distribution and mean particle sizes of 2–3 nm. The NP synthesis was achieved using the ‘bottom‐up’ approach starting from hydrophilic mono‐ and dinuclear Au(I) NHC complexes. The NPs are both stable in buffered aqueous solution and in the presence of the intracellular reducing agent GSH as well as of the serum protein BSA. Characterization by various methods showed important differences between the two colloids depending on the starting ligand and Au(I) carbenic species: the NPs originated from the bidentate NHC ligand, **AuNP‐2**, contain a higher amount of Au(I) and feature a higher ligand surface coverage than **AuNP‐1**. These results suggest that Au(I) NHC molecular species are still prominent in the colloids’ surface for **AuNP‐2**.

After comprehensive characterization, possible applications of the NHC@AuNPs in catalysis were investigated. Therefore, the unsupported Au nanoparticles were tested for the catalytic reduction of nitrophenol substrates and showed high catalytic activity, comparable with analogous Au catalytic systems. The same trend of reactivity was observed for the resazurin reduction. This result is important for future development of AuNP‐templated bioorthogonal transformations in biological/aqueous environment. It is worth mentioning that recent reports explore both molecular gold compounds and AuNPs for a number of metal‐mediated reactions in living systems, specifically aiming at producing novel chemical tools and therapeutic agents.^[32]^

Moreover, the NHC@AuNPs were immobilized on TiO_2_ as the desired support to improve catalyst stability. In general, the immobilization of the Au nanoparticles was low. Interestingly, the resulting supported NPs showed markedly different sizes and catalytic activity. In particular, the **AuNP‐2/TiO_2_** catalyst with only 7 % loading showed appreciable catalytic performance, possibly due to the small NPs size (1.7±0.4 nm) and despite the presence of Au(I) species on the surface as confirmed by XPS. Finally, preliminary studies on the PTT applicability of the NHC@AuNPs were performed in human PC‐3 cancer cells, showing that, despite their small size, these nanoparticles possess selective cancer cell‐killing activity only upon laser irradiation.

In the future, we will focus our attention to improve the experimental protocols for the immobilization of the Au nanoparticles on supports, to understand the nature of chemical treatment to control AuNP size and dispersion, as well as to enhance Au‐support interaction and finally increase the yield of formation of metallic nanoparticles during the synthesis. Moreover, for biomedical applications, tuning of the NPs water‐solubility properties could be achieved using different types of hydrophilic NHCs,^[33]^ including those based on amino acids,^[34]^ whose Au(I) complexes have already the tendency to form AuNPs in aqueous solution.

Recently, multidentate hydrophilic NHC ligands have been used to successfully stabilize larger AuNPs (ca. 10 nm),^[35]^ and could be explored in Au(I) complexes’ scaffold for ‘bottom‐up’ synthetic approaches. Noteworthy, the resilience of the NHC−Au bond allows for multi‐step post‐synthetic modification,^[5a]^ which in the future could be exploited to enable bioconjugation to targeting molecules to improve the selectivity and efficiency of PTT in cancer cells. Overall, our results contribute new knowledge in the field of nanomaterials for applications in catalysis or medicine.

## Experimental Section

### General

Solvents and reagents (reagent grade) were all commercially available and used without further purification. ^1^H and ^13^C{^1^H} NMR spectra were recorded in D_2_O solution on Bruker Avance (400‐500 MHz) NMR spectrometers. UV‐Vis spectra were recorded on a Cary 60 UV‐Vis spectrometer from Aglient Technologies. Fourier‐transform infrared attenuated total reflectance (FTIR‐ATR) spectroscopy was carried out on a Shimadzu IRAffinity‐1S FTIR spectrophotometer equipped with an ATR unit. TG measurements were performed on a PerkinElmer Pyris TGA. XPS was performed on a Thermo K‐alpha^+^ X‐ray photoelectron spectrometer using microfocused Al kα radiation operating at 72 W (6 ma×12 kV) using the 400 micron analysis mode. Charge compensation was achieved using a combination of low energy electrons and argon ion. Data was collected at pass energies of 40 and 150 eV for high resolution and survey data, with step sizes of 0.1 and 1 eV respectively. Data quantification was performed using CasaXPS^[36]^ software Version 2.3.24, using a Shirley type background, and Scofiled sensitivity factors with a TPP‐2 M energy dependence for the photoelectrons. TEM images were obtained using a JEOL JEM 2100 Transmission Electron Microscope operating at 200 kV. Samples were suspended in acetone and dispersed over 300 mesh Cu grids coated with holey carbon film. The images were then analyzed using ImageJ software.^[37]^ The number of NPs analyzed from the TEM images of each sample can be found in the brackets: **AuNP‐1** (391), **AuNP‐2** (507), **AuNP‐1/TiO_2_** (116) and **AuNP‐2/TiO_2_** (226). ICP‐MS analysis was performed on a Agilent 7900 ICP‐MS with I‐AS Autosampler using ^159^Tb as the internal standard element and five‐point calibrations at blank, 1, 10, 100 and 1000 μ g/l using Certified Reference Multi‐Element Calibration Standard 4 from Perkin Elmer (PEN9300234) along with the Certified Internal Standard mix from Agilent (5188–6525). Fluorescence spectroscopy measurements were recorded on a Cary Eclipse Fluorescence Spectrophotometer. Ligands **NHC‐L1** ^[14a]^ and **NHC‐L2**,^[14b]^ and the Au(I) complexes **AuNHC‐1** ^[11]^ and **AuNHC‐2** ^[10]^ were synthesized with minor modifications of literature procedures.

### NHC@AuNP synthesis

The ‘bottom‐up’ method was employed to form the gold nanoparticles **AuNP‐1** and **AuNP‐2**. Each Au(I) NHC complex, **AuNHC‐1** or **AuNHC‐2** (50 mg, 1 equiv), was dissolved in deionized H_2_O (5 mL) at room temperature under stirring. A freshly prepared aqueous NaBH_4_ solution (10 equiv, 1 mL) was added quickly resulting in a darkening of the solution. Samples were left under stirring for 24 h at room temperature. The resulting aqueous solutions of AuNPs were then purified by dialysis using treated cellulose dialysis membrane (Sigma‐Aldrich, D9652) with a molecular weight cut‐off of 14,000 Da.

### AuNP‐1

^1^H NMR (500 MHz, D_2_O) δ 7.21 (d, *J*=1.9 Hz, 2H), 7.19 (d, *J*=1.9 Hz, 2H), 4.28 (t, *J*=6.7 Hz, 4H), 4.16 (t, *J*=6.8 Hz, 4H), 2.84–2.79 (m, 4H), 2.31–2.22 (m, 4H), 1.85–1.76 (m, 4H), 1.29–1.18 (m, 4H), 0.83 (t, *J*=7.4 Hz, 6H).

^13^C{^1^H} NMR (126 MHz, D_2_O) δ 182.90, 121.72, 121.35, 50.62, 49.09, 47.78, 32.78, 26.32, 19.06, 12.88.

### AuNP‐2

^1^H NMR (500 MHz, D_2_O) δ 7.72 (d, *J*=2.2 Hz, 1H), 7.66 (d, *J*=2.1 Hz, 1H), 7.63 (d, *J*=2.2 Hz, 1H), 7.60 (d, *J*=2.2 Hz, 1H), 7.56 (d, *J*=1.3 Hz, 1H), 7.48 (d, *J*=2.1 Hz, 1H), 7.46 (d, *J*=2.1 Hz, 7H), 7.41 (d, *J*=2.1 Hz, 1H), 7.31 (d, *J*=2.0 Hz, 13H), 6.92 (d, *J*=14.0 Hz, 6H), 6.65–6.53 (m, 10H), 6.10 (d, *J*=14.1 Hz, 6H), 4.42–4.20 (m, 10H), 2.90–2.73 (m, 11H), 2.32–2.16 (m, 8H).

^13^C{^1^H} NMR (126 MHz, D_2_O) δ 183.20, 122.81, 121.33, 62.98, 49.88, 47.56, 26.18.

### Stability studies by UV‐visible absorption spectroscopy

Stability studies were conducted by UV‐Vis absorption spectroscopy using solutions of **AuNP‐1** or **AuNP‐2** in MilliQ water, PBS 1x (pH 7.4) and PBS 1x+2 mM GSH or 17 mM BSA. An amount of NHC@AuNPs was dissolved in solution to achieve an absorbance of ca. 0.8 r.u. for the SPR band. Spectra were recorded between 330–1000 nm over time.

### Kinetic study of 2‐, 3‐ and 4‐nitrophenol reduction reactions

Nitrophenol reduction reactions were monitored by UV‐Vis spectroscopy recording spectra between 300–820 nm with an average time of 0.1 s, interval of 1 nm and scan rate of 600 nm/min. Firstly, 100 μ L of a 4‐nitrophenol aqueous solution (3 mM) was added to the cuvette containing the NHC@AuNPs and 1.9 mL of MilliQ water. This was followed by the quick addition of 1 mL of a freshly prepared NaBH_4_ aqueous solution (30 mM) to the cuvette whilst starting spectrophotometric analysis. NaBH_4_ was used in excess with respect to the concentrations of 2‐, 3‐ and 4‐nitrophenol to ensure *pseudo*‐first order reactions. The scans were recorded every 30 sec until the 2‐, 3‐ and 4‐nitrophenol peaks had reached a plateau. The TiO_2_ supported AuNPs were also monitored using this method using 2.4 mg of catalyst in each case.

### Kinetic study of the reduction of resazurin

The reduction of resazurin was monitored by fluorescence spectroscopy following an established experimental protocol.^[38]^ In detail, 10 μ L of a resazurin aqueous solution (3 mM) was added to the cuvette containing the NHC@AuNPs (1 mg) in 2 mL of MilliQ water. This was followed by the quick addition of 1 mL of a freshly prepared NH_2_OH aqueous solution (3 mM) to the cuvette whilst starting the analysis. The substrate reduction was monitored over 1 h with spectra recorded every 30 sec to monitor the fluorescence emission intensity of the product at an excitation wavelength of 532 nm and a slit size of 10 nm for both emission and excitation, respectively.

### Synthesis of supported NHC@AuNPs/TiO_2_

The titania supported NHC@AuNPs were formed by dissolving **AuNP‐1** (7.5 mg) and **AuNP‐2** (7.6 mg) respectively, in MilliQ water (30 mL) whilst stirring before the addition of the titania (**AuNP‐1**: 75 mg and **AuNP‐2**: 76 mg). The mixture was then left to stir overnight at room temperature before filtering. The NPs were then washed with water and left to dry in air overnight.

### Photothermal performance evaluation

The photothermal performance of **AuNP‐2** was evaluated under NIR laser irradiation, performed by an 808 nm semiconductor power‐adjustable laser (808 nm, 340 mW/cm^2^, CNI laser, China). The photothermal conversion efficiency was studied measuring the temperature increase profile of a solution of **AuNP‐2** in PBS buffer (pH 7.4) by a thermocouple upon NIR laser irradiation over a period of 10 min and a 20 min cooling period. The photothermal efficiency was calculated using Equations (1)–(3)^[39]^.

(1) $$\eta =\frac{\mathrm{hS}\left(T_{\max}-T_{\mathrm{sur}}\right)-Q_{\mathrm{Dis}}}{I\left(1-10^{-A_{808}}\right)}$$

(2) $$t=-\tau \ln\left(\frac{T-T_{\mathrm{sur}}}{T_{\max}-T_{\mathrm{sur}}}\right)$$

(3) $$\tau =\frac{{\mathrm{mC}}_{p}}{\mathrm{hS}}$$

### Cytotoxicity and photothermal therapy performance evaluation

Human prostate adenocarcinoma cells (cell line PC‐3, American Type Culture Collection, Rockville, MD) were firstly uniformly seeded into a 96‐well plate with ca. 3000 cells/well and allowed to grow for 24 h before AuNPs co‐culturing and/or laser treatment. In the 96‐well plate, the wells with cells were divided into different experimental groups (n=3) and were co‐cultured with 0.1, 0.2 and 0.4 mg/mL of **AuNP‐2**, respectively. The blank group was treated with normal growth medium and diluted by DPBS buffer. The toxicity of the nanoparticles and PTT therapy were evaluated using the MTT assay (3‐(4,5‐dimethylthiazol‐2‐yl)‐2,5‐diphenyltetrazolium bromide, Sigma, M5655‐100 mg). For the toxicity studies, the cells were co‐cultured with the **AuNP‐2** in the dark with 5 % CO_2_ at 37 °C for 24 h before the MTT assay evaluation. For the photothermal therapy studies, prior to the laser treatment, PC‐3 cells were incubated in the dark with 5 % CO_2_ at 37 °C for 6 h. Afterwards, cells were irradiated using the NIR laser for 10 min (808 nm, 340 mW/cm^2^). After a further 18 h incubation, the antiproliferative effect of photothermal therapy was evaluated using the MTT assay.

## Conflict of interest

The authors declare no conflict of interest.

1

## Supporting information

As a service to our authors and readers, this journal provides supporting information supplied by the authors. Such materials are peer reviewed and may be re‐organized for online delivery, but are not copy‐edited or typeset. Technical support issues arising from supporting information (other than missing files) should be addressed to the authors.

Supporting Information

## Data Availability

The data that support the findings of this study are available in the supplementary material of this article.

## References

[1a] M. C. M. Daniel , D. Astruc , Chem. Rev. 2004, 104, 293–346; 14719978 10.1021/cr030698+

[1b] K. Saha , S. S. Agasti , C. Kim , X. Li , V. M. Rotello , Chem. Rev. 2012, 112, 2739–2779; 22295941 10.1021/cr2001178 PMC4102386

[1c] W. Zhou , X. Gao , D. Liu , X. Chen , Chem. Rev. 2015, 115, 10575–10636; 26114396 10.1021/acs.chemrev.5b00100 PMC5226399

[1d] I. Venditti , Materials 2017, 10, 97; 28772458 10.3390/ma10020097 PMC5459143

[1e] G. J. Hutchings , ACS Cent. Sci. 2018, 4, 1095–1101. 30276242 10.1021/acscentsci.8b00306 PMC6161050

[2] M. Grzelczak , J. Perez-Juste , P. Mulvaney , L. M. Liz-Marzan , Chem. Soc. Rev. 2008, 37, 1783–1791. 18762828 10.1039/b711490g

[3a] C. Eisen , J. M. Chin , M. R. Reithofer , Chem. Asian J. 2021, 16, 3026–3037; 34399027 10.1002/asia.202100731 PMC8597167

[3b] S. R. Thomas , A. Casini , J. Organomet. Chem. 2021, 938, 121743;

[3c] C. A. Smith , M. R. Narouz , P. A. Lummis , I. Singh , A. Nazemi , C. H. Li , C. M. Crudden , Chem. Rev. 2019, 119, 4986–5056; 30938514 10.1021/acs.chemrev.8b00514

[3d] Y. Y. An , J. G. Yu , Y. F. Han , Chin. J. Chem. 2018, 37, 76–87;

[3e] S. Engel , E. C. Fritz , B. J. Ravoo , Chem. Soc. Rev. 2017, 46, 2057–2075; 28272608 10.1039/c7cs00023e

[3f] S. Roland , X. Ling , M. P. Pileni , Langmuir 2016, 32, 7683–7696; 27412075 10.1021/acs.langmuir.6b01458 PMC4980691

[3g] H. Shen , G. Tian , Z. Xu , L. Wang , Q. Wu , Y. Zhang , B. K. Teo , N. Zheng , Coord. Chem. Rev. 2022, 458.

[4] Q. Tang , D.-e. Jiang , Chem. Mater. 2017, 29, 6908–6915.

[5a] J. F. DeJesus , L. M. Sherman , D. J. Yohannan , J. C. Becca , S. L. Strausser , L. F. P. Karger , L. Jensen , D. M. Jenkins , J. P. Camden , Angew. Chem. Int. Ed. 2020, 59, 7585–7590; 10.1002/anie.202001440 32092219

[5b] A. Ferry , K. Schaepe , P. Tegeder , C. Richter , K. M. Chepiga , B. J. Ravoo , F. Glorius , ACS Catal. 2015, 5, 5414–5420;

[5c] M. J. MacLeod , J. A. Johnson , J. Am. Chem. Soc. 2015, 137, 7974–7977; 26081724 10.1021/jacs.5b02452

[5d] K. Salorinne , R. W. Y. Man , C. H. Li , M. Taki , M. Nambo , C. M. Crudden , Angew. Chem. Int. Ed. 2017, 56, 6198–6202; 10.1002/anie.201701605 28407403

[5e] P. Tegeder , M. Freitag , K. M. Chepiga , S. Muratsugu , N. Moller , S. Lamping , M. Tada , F. Glorius , B. J. Ravoo , Chem. Eur. J. 2018, 24, 18682–18688; 30246891 10.1002/chem.201803274

[5f] A. J. Young , C. Eisen , G. Rubio , J. M. Chin , M. R. Reithofer , J. Inorg. Biochem. 2019, 199, 110707; 31369908 10.1016/j.jinorgbio.2019.110707

[5g] G. Fernández , L. Bernardo , A. Villanueva , R. Pleixats , New J. Chem. 2020, 44, 6130–6141.

[6] R. W. Y. Man , C. H. Li , M. W. A. MacLean , O. V. Zenkina , M. T. Zamora , L. N. Saunders , A. Rousina-Webb , M. Nambo , C. M. Crudden , J. Am. Chem. Soc. 2018, 140, 1576–1579. 29211456 10.1021/jacs.7b08516

[7] M. J. MacLeod , A. J. Goodman , H. Z. Ye , H. V. Nguyen , T. Van Voorhis , J. A. Johnson , Nat. Chem. 2019, 11, 57–63. 30420777 10.1038/s41557-018-0159-8

[8] G. Wang , A. Ruhling , S. Amirjalayer , M. Knor , J. B. Ernst , C. Richter , H. J. Gao , A. Timmer , H. Y. Gao , N. L. Doltsinis , F. Glorius , H. Fuchs , Nat. Chem. 2017, 9, 152–156. 28282049 10.1038/nchem.2622

[9] W. Yang , H. Liang , S. Ma , D. Wang , J. Huang , Sustain. Mater. Technol. 2019, 22.

[10] O. Karaca , V. Scalcon , S. M. Meier-Menches , R. Bonsignore , J. Brouwer , F. Tonolo , A. Folda , M. P. Rigobello , F. E. Kuhn , A. Casini , Inorg. Chem. 2017, 56, 14237–14250. 29095609 10.1021/acs.inorgchem.7b02345

[11] A. Almássy , C. E. Nagy , A. C. Bényei , F. Joó , Organometallics 2010, 29, 2484–2490.

[12] E. A. Baquero , S. Tricard , J. C. Flores , E. de Jesus , B. Chaudret , Angew. Chem. Int. Ed. 2014, 53, 13220–13224; 10.1002/anie.201407758 25267410

[13] M. O. N. v. d. L'Isle , M. C. Ortega-Liebana , A. Unciti-Broceta , Curr. Opin. Chem. Biol. 2021, 61, 32–42. 33147552 10.1016/j.cbpa.2020.10.001

[14a] D. Prajapati , C. Schulzke , M. K. Kindermann , A. R. Kapdi , RSC Adv. 2015, 5, 53073–53085;

[14b] G. Papini , M. Pellei , G. G. Lobbia , A. Burini , C. Santini , Dalton Trans. 2009, 6985–6990. 20449140 10.1039/b906994a

[15] P. K. Ngumbi , S. W. Mugo , J. M. Ngaruiya , IOSR J. Appl. Chem. 2018, 11, 25–29.

[16] T. Rajkumar , G. Ranga Rao , Mater. Chem. Phys. 2008, 112, 853–857.

[17a] M. Brust , M. Walker , D. Bethell , D. J. Schiffrin , R. Whyman , J. Chem. Soc. Chem. Commun. 1994, 801–802;

[17b] L. Prati , A. Villa , Acc. Chem. Res. 2014, 47, 855–863. 24266851 10.1021/ar400170j

[18a] M. R. Narouz , C. H. Li , A. Nazemi , C. M. Crudden , Langmuir 2017, 33, 14211–14219; 29148789 10.1021/acs.langmuir.7b02248

[18b] A. J. Young , M. Sauer , G. Rubio , A. Sato , A. Foelske , C. J. Serpell , J. M. Chin , M. R. Reithofer , Nanoscale 2019, 11, 8327–8333. 30984947 10.1039/c9nr00905a

[19] N. Bridonneau , L. Hippolyte , D. Mercier , D. Portehault , M. Desage-El Murr , P. Marcus , L. Fensterbank , C. Chaneac , F. Ribot , Dalton Trans. 2018, 47, 6850–6859. 29725678 10.1039/c8dt00416a

[20] V. Alderucci , L. Pino , P. L. Antonucci , W. Roh , J. Cho , H. Kim , D. L. Cocke , V. Antonucci , Mater. Chem. Phys. 1995, 41, 9–14.

[21] R. Ye , A. V. Zhukhovitskiy , R. V. Kazantsev , S. C. Fakra , B. B. Wickemeyer , F. D. Toste , G. A. Somorjai , J. Am. Chem. Soc. 2018, 140, 4144–4149. 29506380 10.1021/jacs.8b01017

[22] K. Layek , M. L. Kantam , M. Shirai , D. Nishio-Hamane , T. Sasaki , H. Maheswaran , Green Chem. 2012, 14.

[23] P. Zhao , N. Li , D. Astruc , Coord. Chem. Rev. 2013, 257, 638–665.

[24] S. M. Rogers , C. R. A. Catlow , D. Gianolio , P. P. Wells , N. Dimitratos , Faraday Discuss. 2018, 208, 443–454. 29796530 10.1039/c7fd00216e

[25a] Z. Jiang , W. Zhang , L. Jin , X. Yang , F. Xu , J. Zhu , W. Huang , J. Phys. Chem. C 2007, 111, 12434–12439;

[25b] P. Konova , A. Naydenov , C. Venkov , D. Mehandjiev , D. Andreeva , T. Tabakova , J. Mol. Catal. A 2004, 213, 235–240.

[26] M. Flytzani-Stephanopoulos , Acc. Chem. Res. 2014, 47, 783–792. 24266870 10.1021/ar4001845

[27] K. Morgan , R. Burch , M. Daous , J. J. Delgado , A. Goguet , C. Hardacre , L. A. Petrov , D. W. Rooney , Catal. Sci. Technol. 2014, 4.

[28] Q. Fu , H. Saltsburg , M. Flytzani-Stephanopoulos , Science 2013, 301, 935–938. 10.1126/science.1085721 12843399

[29a] X. Huang , W. Qian , I. H. El-Sayed , M. A. El-Sayed , Lasers Surg. Med. 2007, 39, 747–753; 17960762 10.1002/lsm.20577

[29b] A. Slablab , L. Le Xuan , M. Zielinski , Y. de Wilde , V. Jacques , D. Chauvat , J.-F. Roch , Opt. Express 2012, 20, 220–227. 22274345 10.1364/OE.20.000220

[30] Y. Feng , Y. Chang , X. Sun , Y. Cheng , R. Zheng , X. Wu , L. Wang , X. Ma , X. Li , H. Zhang , Biomater. Sci. 2019, 7, 1448–1462. 30666994 10.1039/c8bm01122b

[31] W. Yang , B. Xia , L. Wang , S. Ma , H. Liang , D. Wang , J. Huang , Mater. Today Sustain. 2021, 13.

[32] S. R. Thomas , A. Casini , Curr. Opin. Chem. Biol. 2020, 55, 103–110. 32086166 10.1016/j.cbpa.2019.12.007

[33] L. A. Schaper , S. J. Hock , W. A. Herrmann , F. E. Kuhn , Angew. Chem. Int. Ed. 2013, 52, 270–289; 10.1002/anie.201205119 23143709

[34a] C. H. G. Jakob , B. Dominelli , E. M. Hahn , T. O. Berghausen , T. Pinheiro , F. Marques , R. M. Reich , J. D. G. Correia , F. E. Kuhn , Chem. Asian J. 2020, 15, 2754–2762; 32592289 10.1002/asia.202000620 PMC7689731

[34b] F. Schmitt , K. Donnelly , J. K. Muenzner , T. Rehm , V. Novohradsky , V. Brabec , J. Kasparkova , M. Albrecht , R. Schobert , T. Mueller , J. Inorg. Biochem. 2016, 163, 221–228. 27491634 10.1016/j.jinorgbio.2016.07.021

[35] N. A. Nosratabad , Z. Jin , L. Du , M. Thakur , H. Mattoussi , Chem. Mater. 2021, 33, 921–933.

[36] N. Fairley , V. Fernandez , M. Richard-Plouet , C. Guillot-Deudon , J. Walton , E. Smith , D. Flahaut , M. Greiner , M. Biesinger , S. Tougaard , D. Morgan , J. Baltrusaitis , Appl. Surf. Sci. 2021, 5.

[37] C. A. Schneider , W. S. Rasband , K. W. Eliceiri , Nat. Methods 2012, 9, 671–675. 22930834 10.1038/nmeth.2089 PMC5554542

[38] X. Zhou , W. Xu , G. Liu , D. Panda , P. Chen , J. Am. Chem. Soc. 2010, 132, 138–146. 19968305 10.1021/ja904307n

[39a] X. Liu , B. Li , F. Fu , K. Xu , R. Zou , Q. Wang , B. Zhang , Z. Chen , J. Hu , Dalton Trans. 2014, 43, 11709–11715; 24950757 10.1039/c4dt00424h

[39b] M. Sun , F. Liu , Y. Zhu , W. Wang , J. Hu , J. Liu , Z. Dai , K. Wang , Y. Wei , J. Bai , W. Gao , Nanoscale 2016, 8, 4452–4457. 26847879 10.1039/c6nr00056h

