# Supplementary material for: Bottom‐up Synthesis of Water‐Soluble Gold Nanoparticles Stabilized by N‐Heterocyclic Carbenes: From Structural Characterization to Applications
Source: Chemistry. 2022 Aug 10;28(56):e202201575. doi: 10.1002/chem.202201575 (PMC9804724; doi:10.1002/chem.202201575)
Supplement: Supplementary file 1 — Supporting Information [file CHEM-28-0-s001.pdf]

Chemistry–A European Journal

Supporting Information

Bottom-up Synthesis of Water-Soluble Gold Nanoparticles Stabilized by N-Heterocyclic Carbenes: From Structural Characterization to Applications

Sophie R. Thomas, Wenjie Yang, David J. Morgan, Thomas E. Davies, Jiao Jiao Li, Roland A. Fischer, Jun Huang, Nikolaos Dimitratos,* and Angela Casini*

Figures

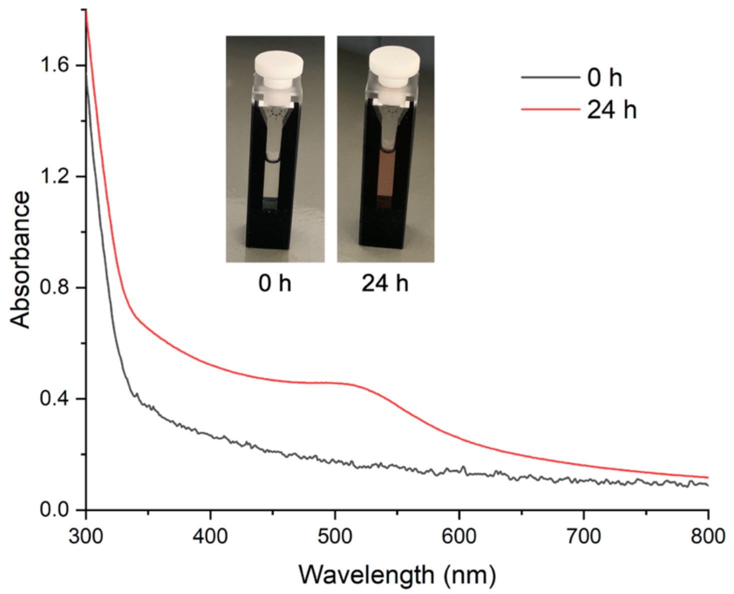

Figure S0. UV-Vis absorption spectra for the *in situ* reduction of the Au(I) complex **AuNHC-2** with 10 eq. of $NaBH_4$ in MilliQ water over 24 h. Insert: Image of **AuNHC-2** solution in cuvette at time 0 and after 24 h.

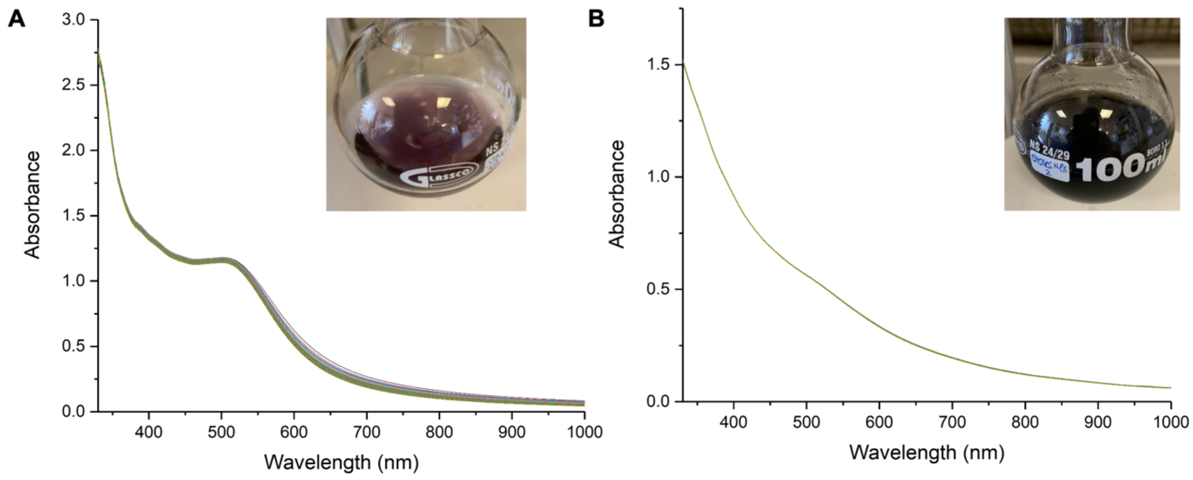

Figure S1. UV-Vis absorption spectra of **A**) **AuNP-1** and **B**) **AuNP-2** in MilliQ water, spectra recorded every 15 min for the first hour and then every 30 min until 15 h. Insert: Images of the NHC@AuNP solutions after reduction.

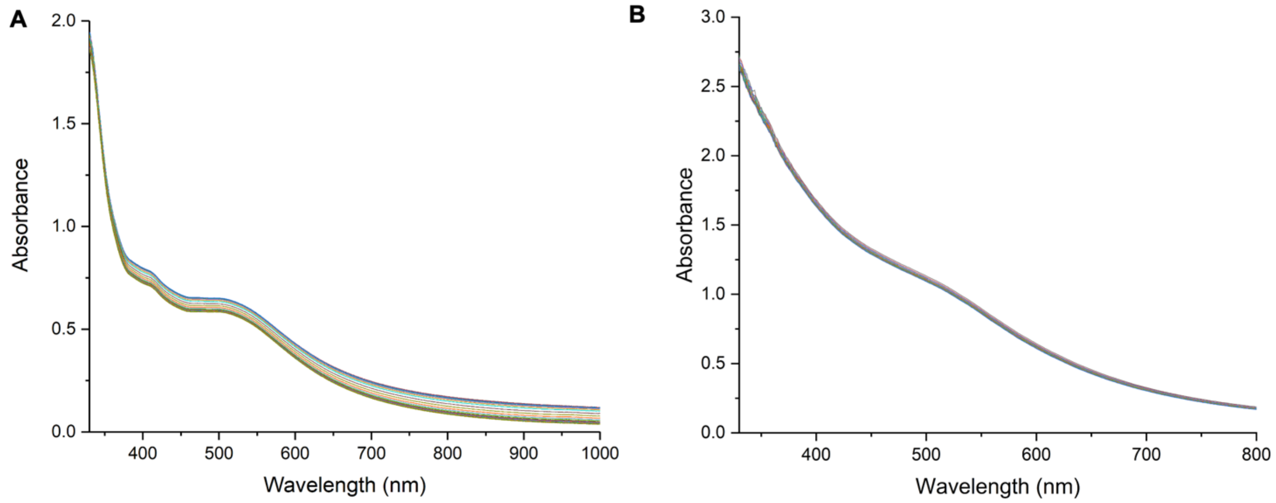

Figure S2. UV-Vis absorption spectra of **A**) **AuNP-1** and **B**) **AuNP-2** in PBS 1x (pH 7.4), spectra recorded every 15 min for the first hour and then every 30 min until 15 h.

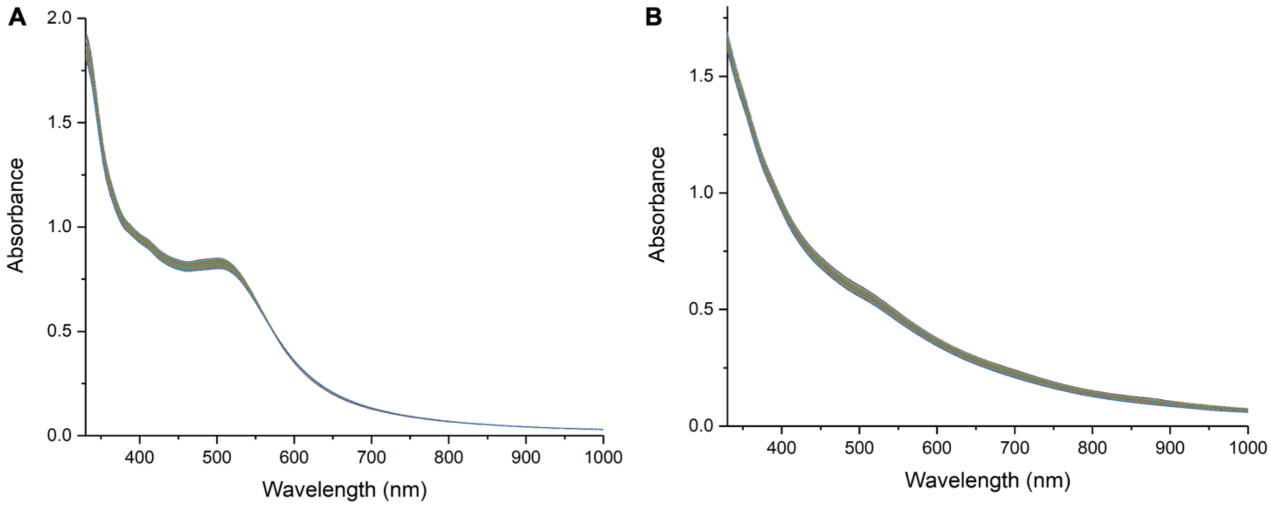

Figure S3. UV-Vis absorption spectra of **A**) **AuNP-1** and **B**) **AuNP-2** in PBS 1x (pH 7.4) + GSH (2 mM), recorded every 10 min for the first hour, then every 30 min until 24 h.

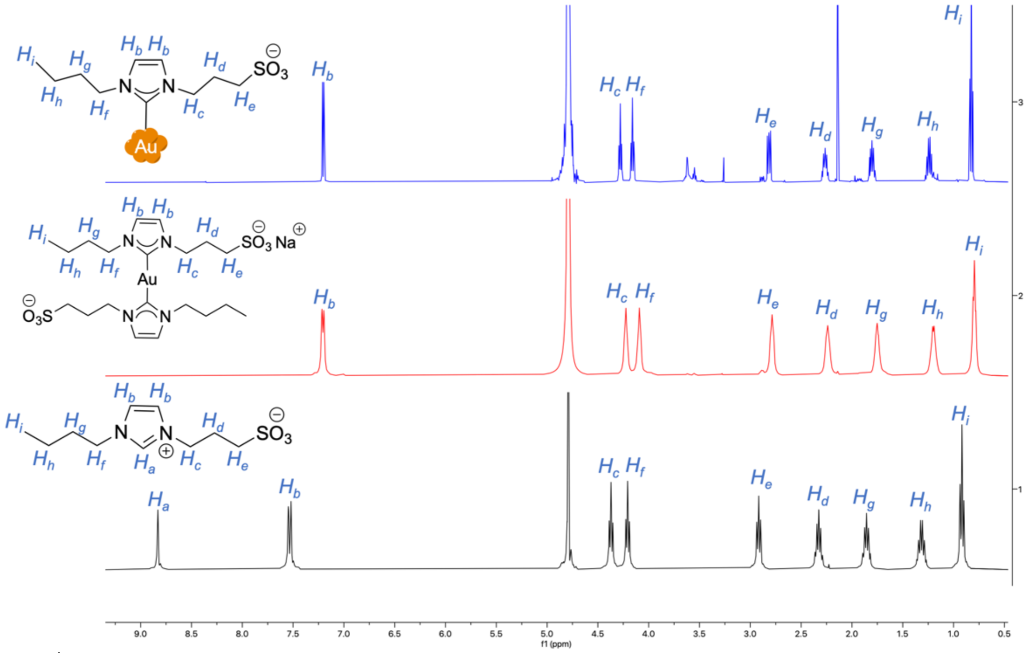

Figure S4. ^{1}H NMR spectra of imidazolium ligand **NHC-1** (bottom, black), complex **AuNHC-1** (middle, red) and **AuNP-1** (top, blue) in D_2O.

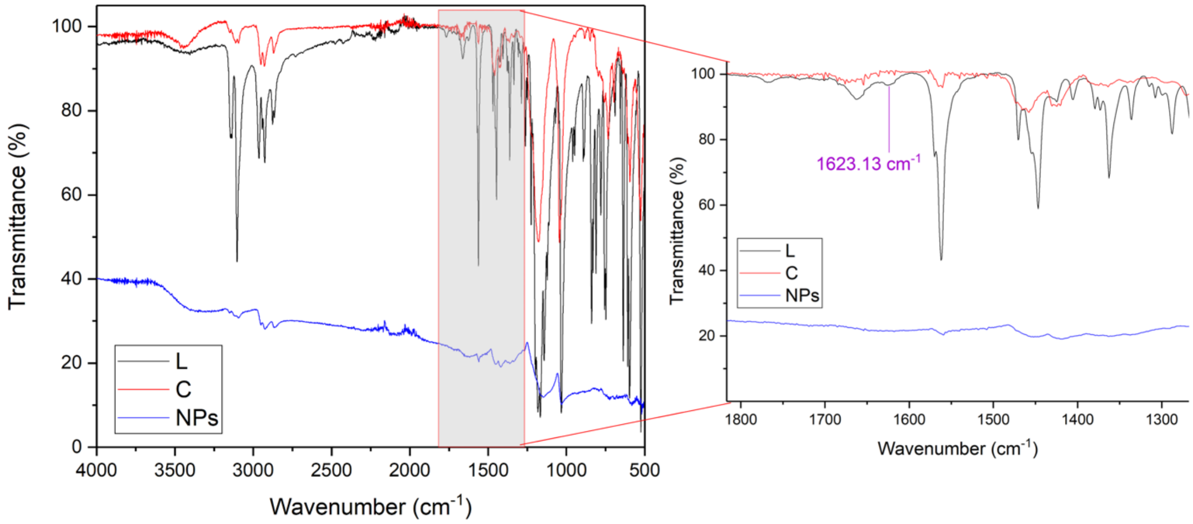

Figure S5. FTIR-ATR spectra of ligand **NHC-1** (L, black trace), complex **AuNHC-1** (C, red trace) and **AuNP-1** (NHC@AuNPs, blue trace); imidazolium C-H ring stretch signal (1623.13 cm^{-1}) only seen in the free imidazolium ligand.

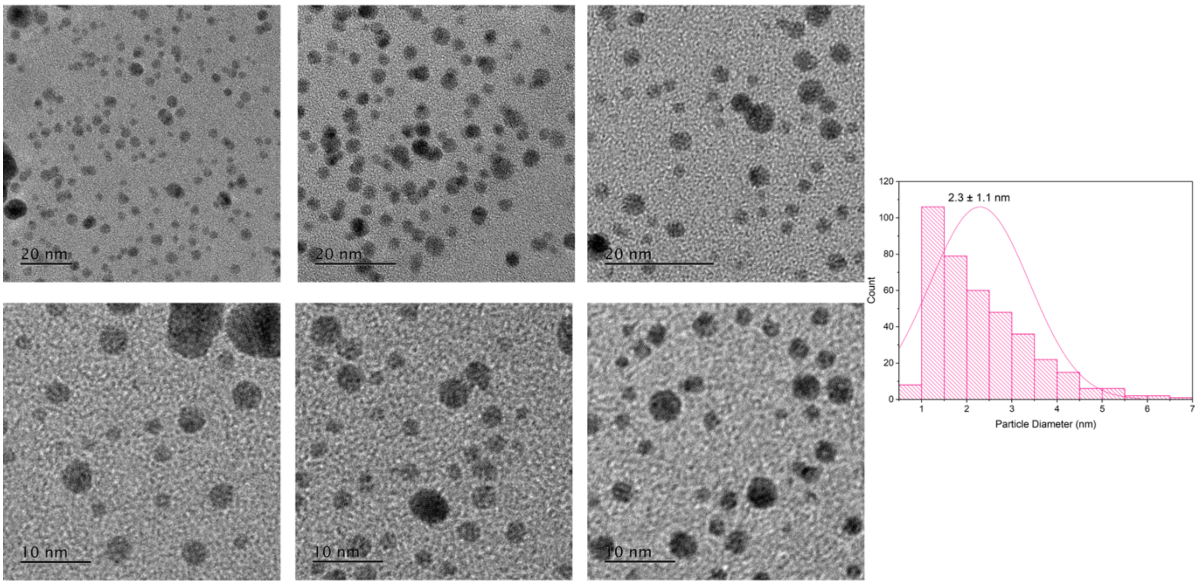

Figure S6. TEM images of **AuNP-1** at different magnifications which were used to obtain the particle size distribution histogram.

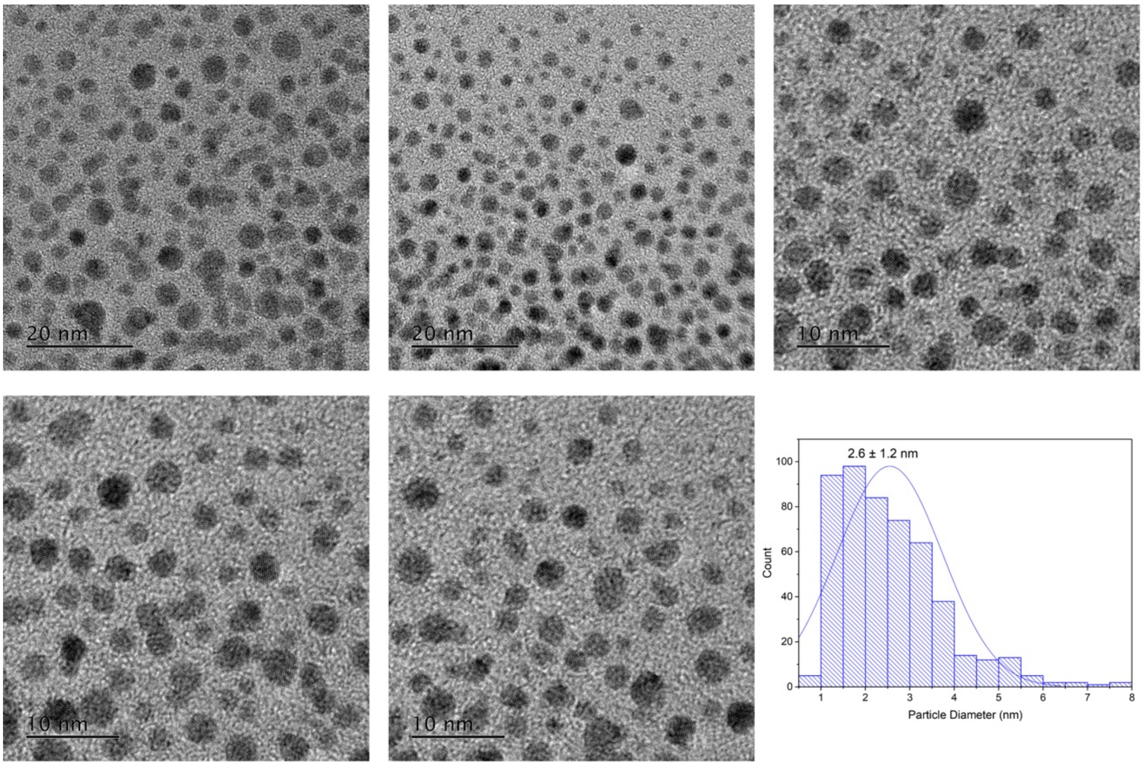

Figure S7. TEM images of **AuNP-2** at different magnifications which were used to obtain the particle size distribution histogram.

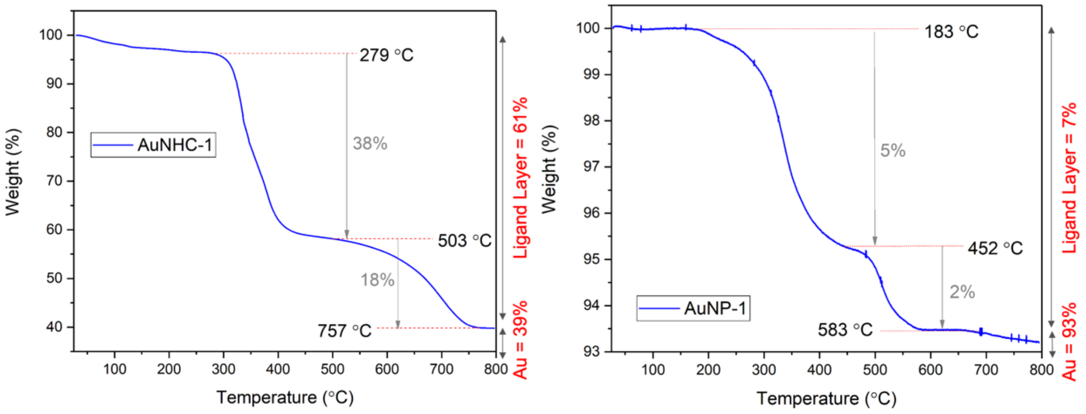

Figure S8. TG curves of **AuNHC-1** (left) and **AuNP-1** (right).

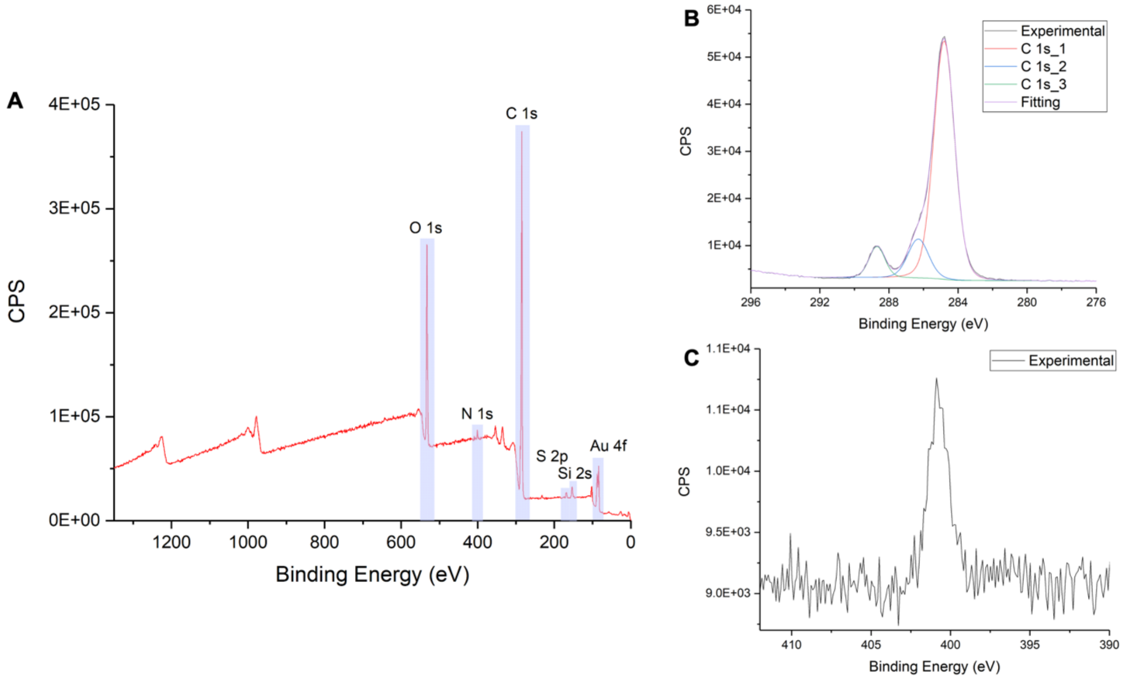

Figure S9. XPS Spectra for **AuNP-1**: **A**) survey spectrum, **B**) experimental spectra and fitting of C 1s with deconvolution of the peaks and **C**) experimental spectrum of N 1s.

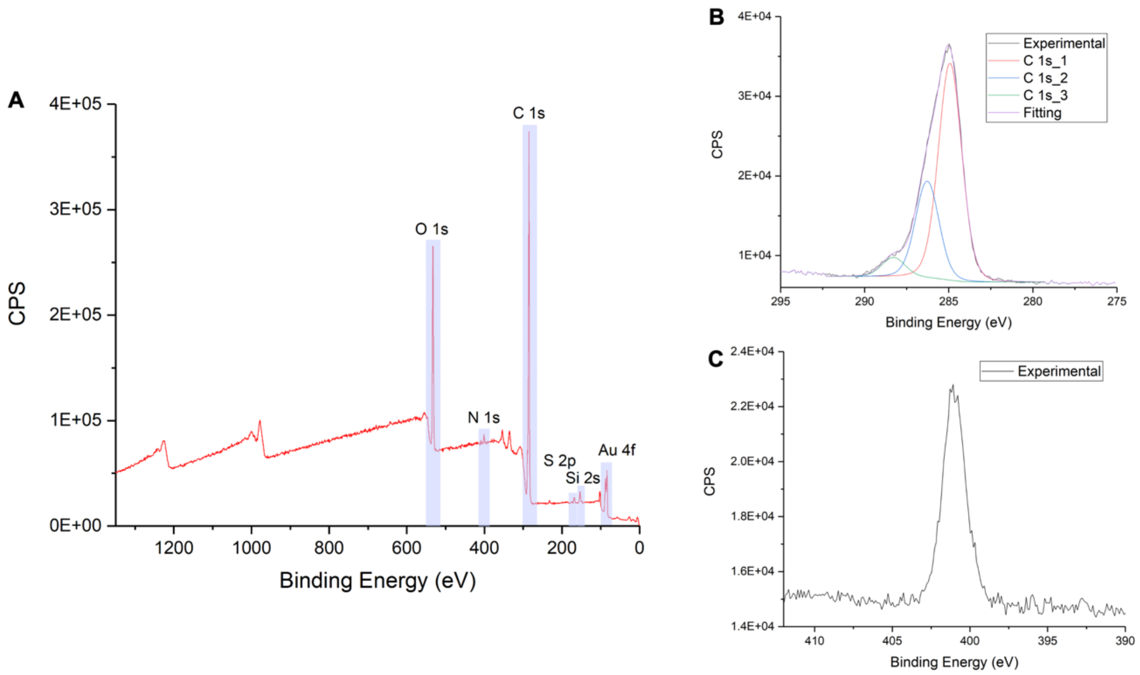

Figure S10. XPS Spectra for **AuNP-2**: **A**) survey spectrum, **B**) experimental spectra and fitting of C 1s with deconvolution of the peaks and **C**) experimental spectrum of N1s.

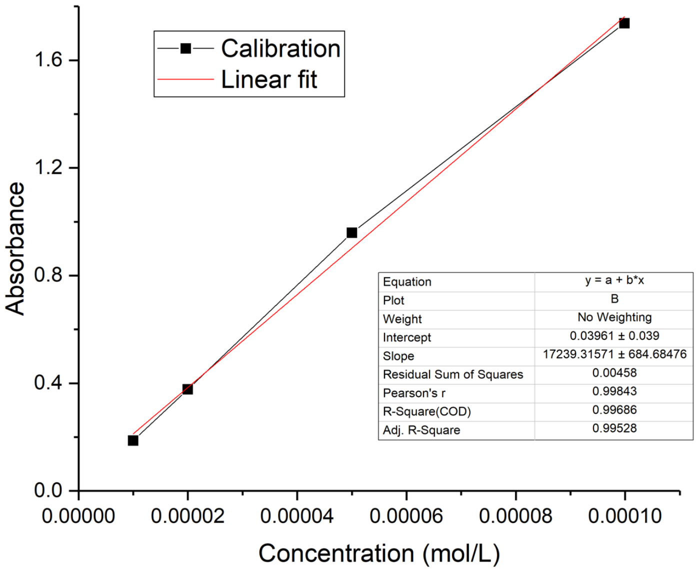

Figure S11. Calibration plot for 4-nitrophenol.

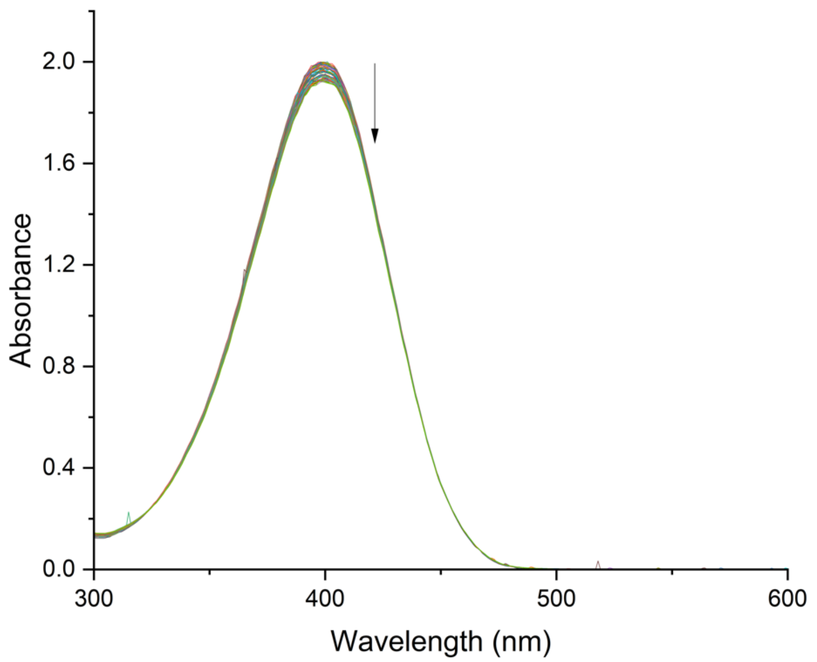

Figure S12. UV-Vis absorption kinetic study for the catalytic reduction of 4-nitrophenol (4-NPhen) by **AuNHC-2** (1 mg) in the presence of 30 mM $NaBH_4$ in MilliQ water at r.t. over ca. 30 min, with spectra recorded every 30 sec over 4 h.

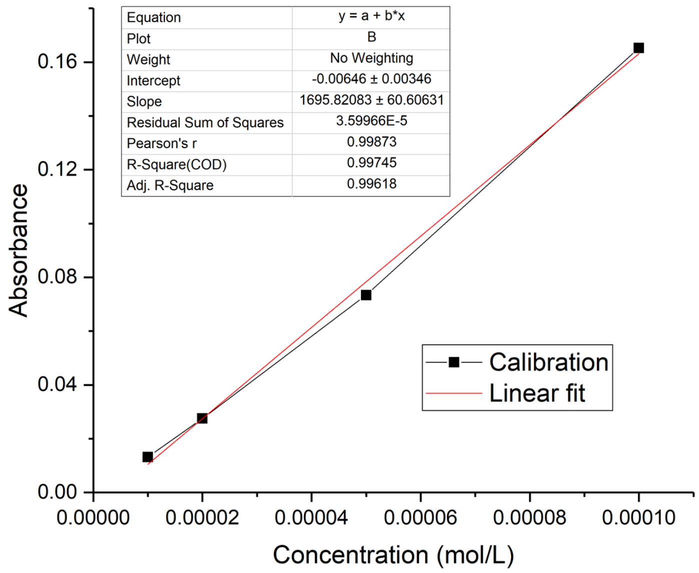

Figure S13. Calibration plot for 3-nitrophenol.

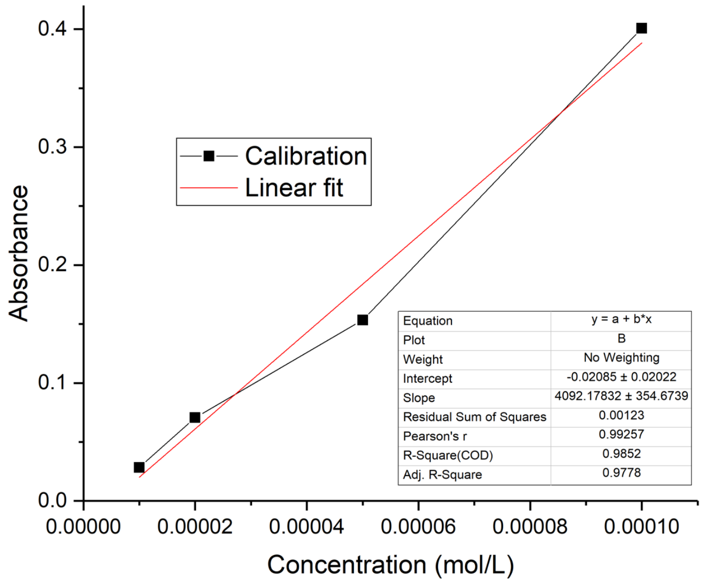

Figure S14. Calibration plot for 2-nitrophenol.

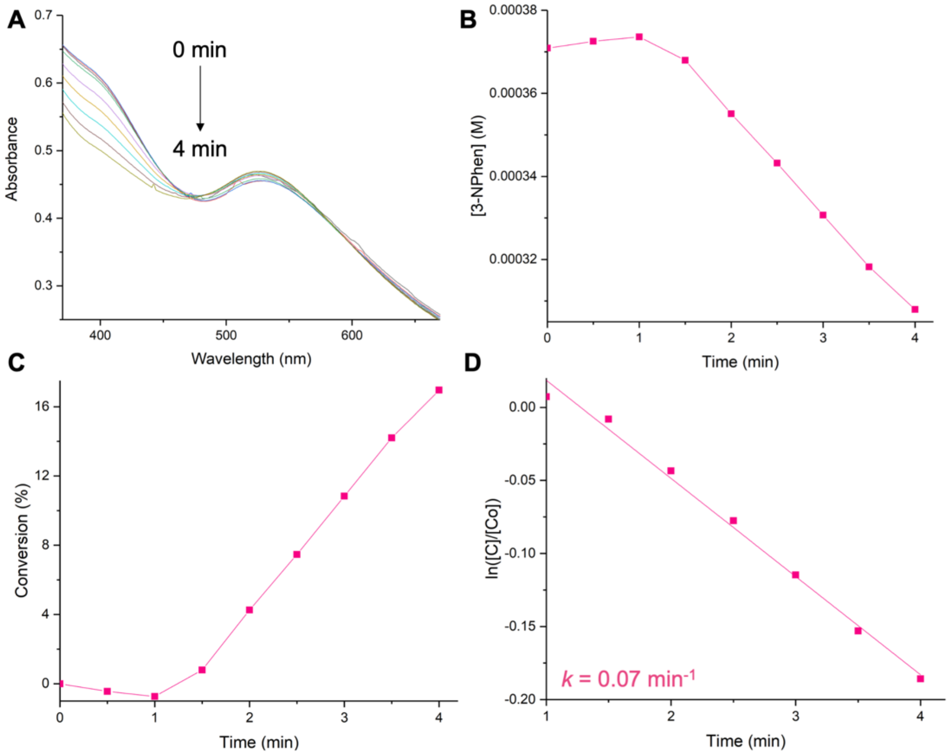

Figure S15. A) UV-Vis absorption kinetic studies for the reduction of 3-nitrophenol (3-NPhen) into 3-aminophenol catalyzed by **AuNP-1** (0.6 mg) in water at r.t. over 4 min, with a spectrum recorded every 30 sec. **B**) Plot of [3-NPhen] *vs*. time monitored at 384 nm. **C**) Plot of % substrate conversion *vs.* time. **D**) Plot of ln([C]/[Co]) *vs*. time for the reduction of 3-nitrophenol showing first order kinetics, and calculated rate constant.

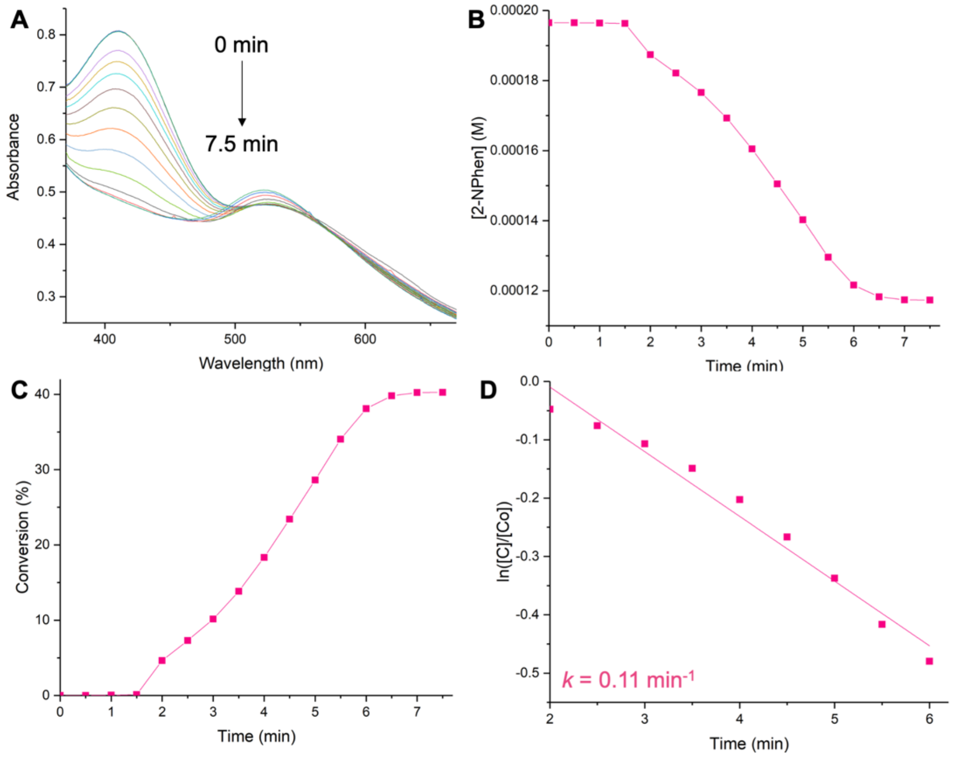

Figure S16. A) UV-Vis absorption kinetic studies for the reduction of 2-nitrophenol (2-NPhen) into 2-aminophenol catalyzed by **AuNP-1** (0.5 mg) in water at r.t. over 7.5 min, with a spectrum recorded every 30 sec. **B**) Plot of [2-NPhen] *vs*. time monitored at 415 nm. **C**) Plot of % substrate conversion *vs.* time. **D**) Plot of ln([C]/[Co]) *vs*. time for the reduction of 2-nitrophenol showing first order kinetics, and calculated rate constant.

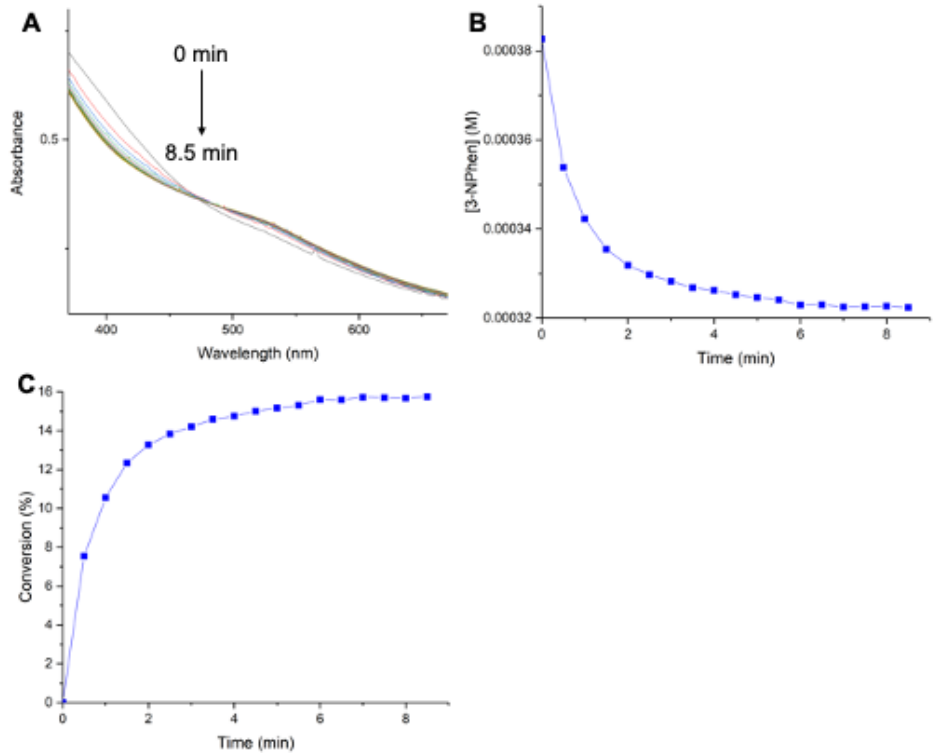

Figure S17. A) UV-Vis absorption kinetic studies for the reduction of 3-nitrophenol (3-NPhen) into 3-aminophenol catalyzed by **AuNP-2** (0.5 mg) in water at r.t. over 8.5 min, with a spectrum recorded every 30 sec. **B**) Plot of [3-NPhen] *vs.* time monitored at 384 nm. **C**) Plot of % substrate conversion *vs.* time.

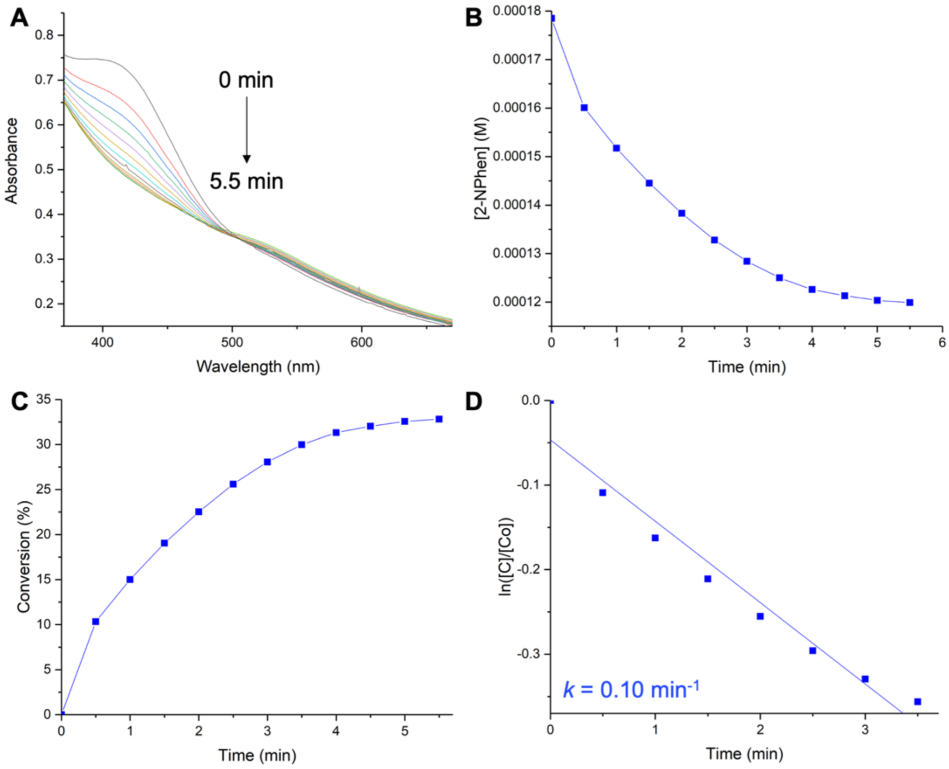

Figure S18. A) UV-Vis absorption kinetic studies for the reduction of 2-nitrophenol (2-NPhen) into 2-aminophenol catalyzed by **AuNP-2** (0.5 mg) in water at r.t. over 5.5 min, with a spectrum recorded every 30 sec. **B**) Plot of [2-NPhen] *vs.* time monitored at 415 nm. **C**) Plot of % substrate conversion *vs.* time. **D**) Plot of ln([C]/[Co]) *vs*. time for the reduction of 2-nitrophenol showing first order kinetics, and calculated rate constant.

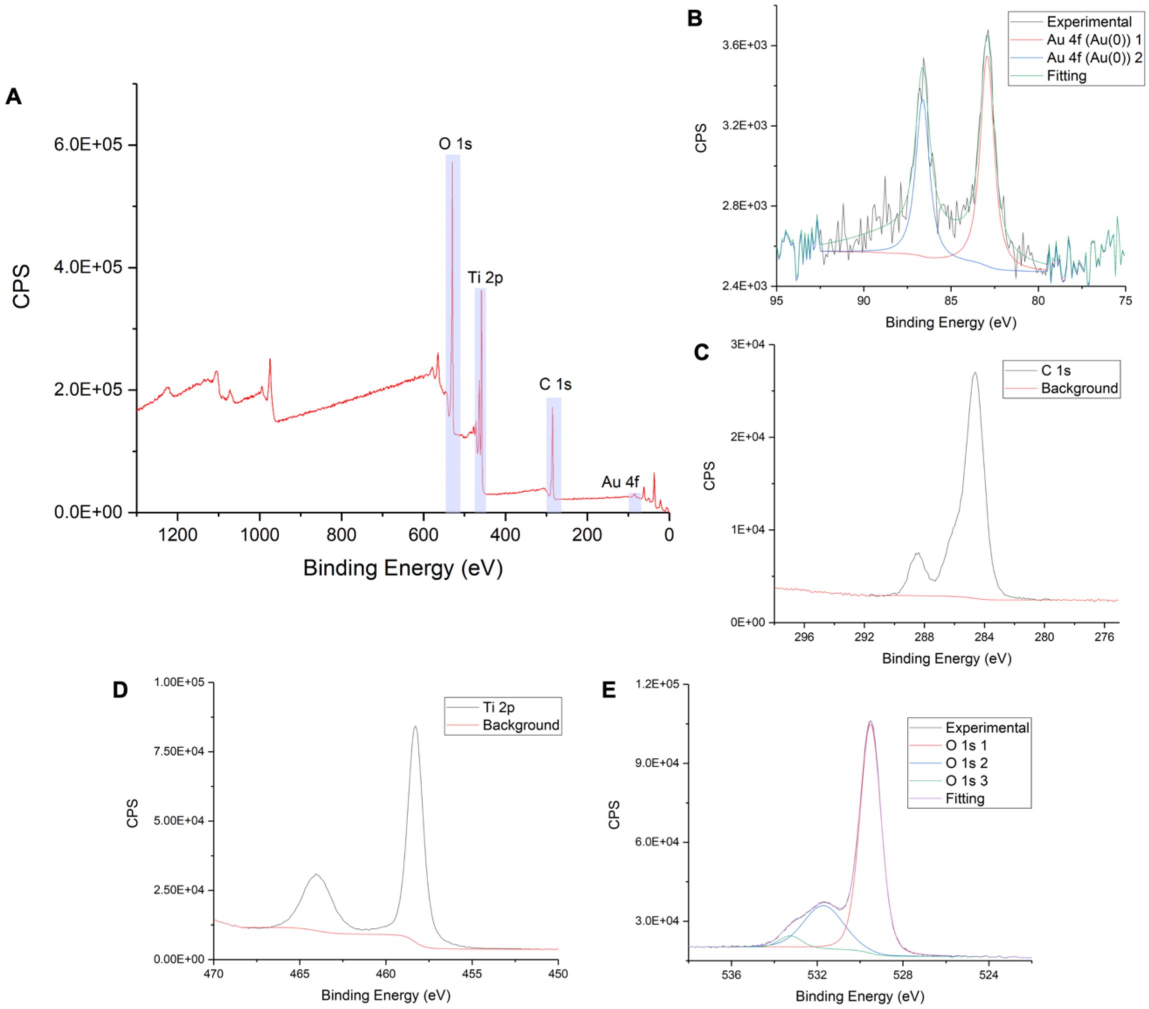

Figure S19. XPS Spectra for **AuNP-1/TiO$_2$**: **A**) survey spectrum, **B**) experimental spectra and fitting of Au 4f with deconvolution of the peaks, **C**) experimental spectra and background of C 1s, **D**) experimental spectra and background of Ti 2p, and **E**) experimental spectra and fitting of O 1s with deconvolution of the peaks.

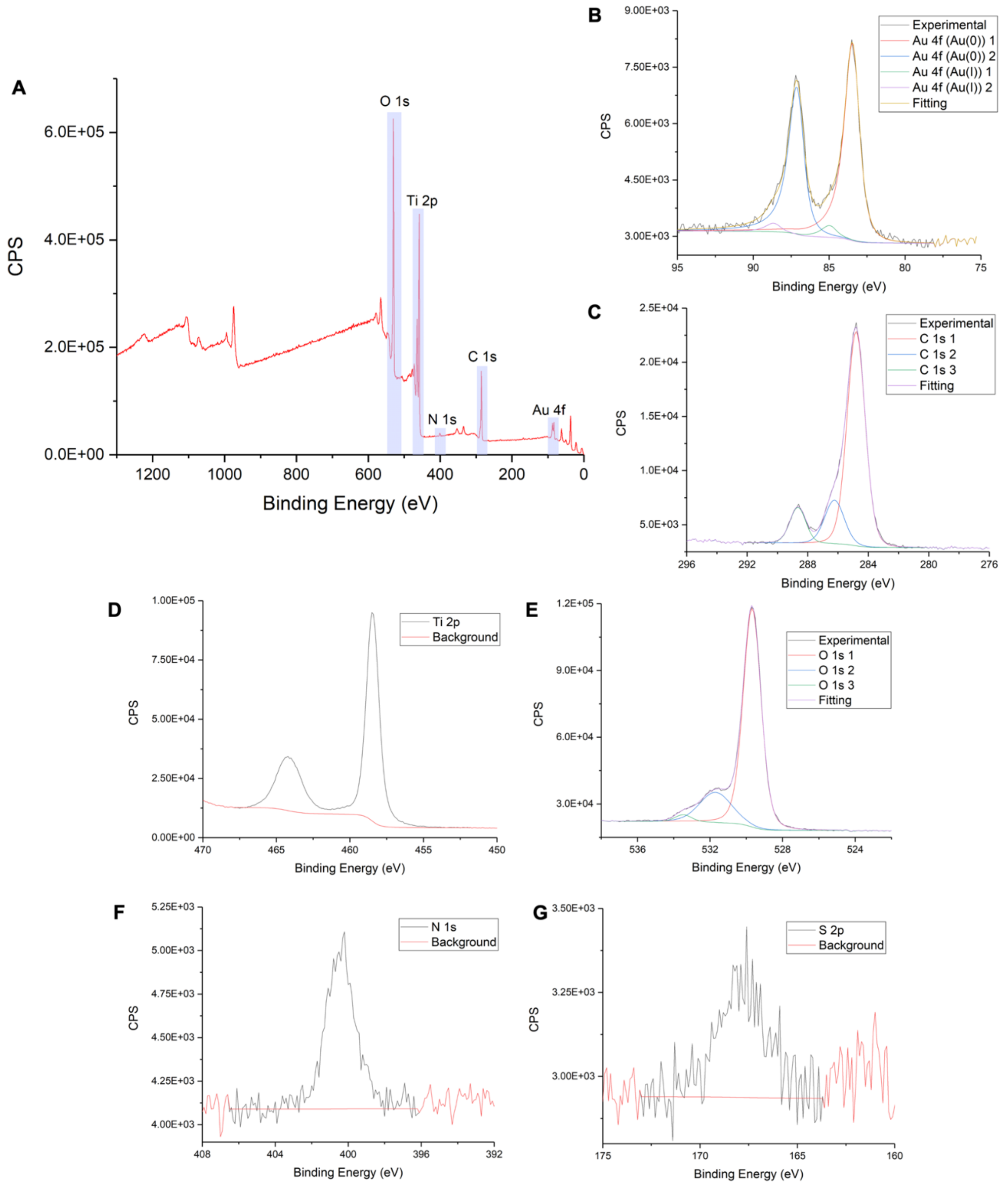

Figure S20. XPS Spectra for **AuNP-2/TiO$_2$**: **A**) survey spectrum, **B**) experimental spectra and fitting of Au 4f with deconvolution of the peaks, **C**) experimental spectra and fitting of C 1s with deconvolution of the peaks, **D**) experimental spectra and background of Ti 2p, **E**) experimental spectra and fitting of O 1s with deconvolution of the peaks, **F**) experimental spectra and background of N 1s and **G**) experimental spectra and background of S 2p.

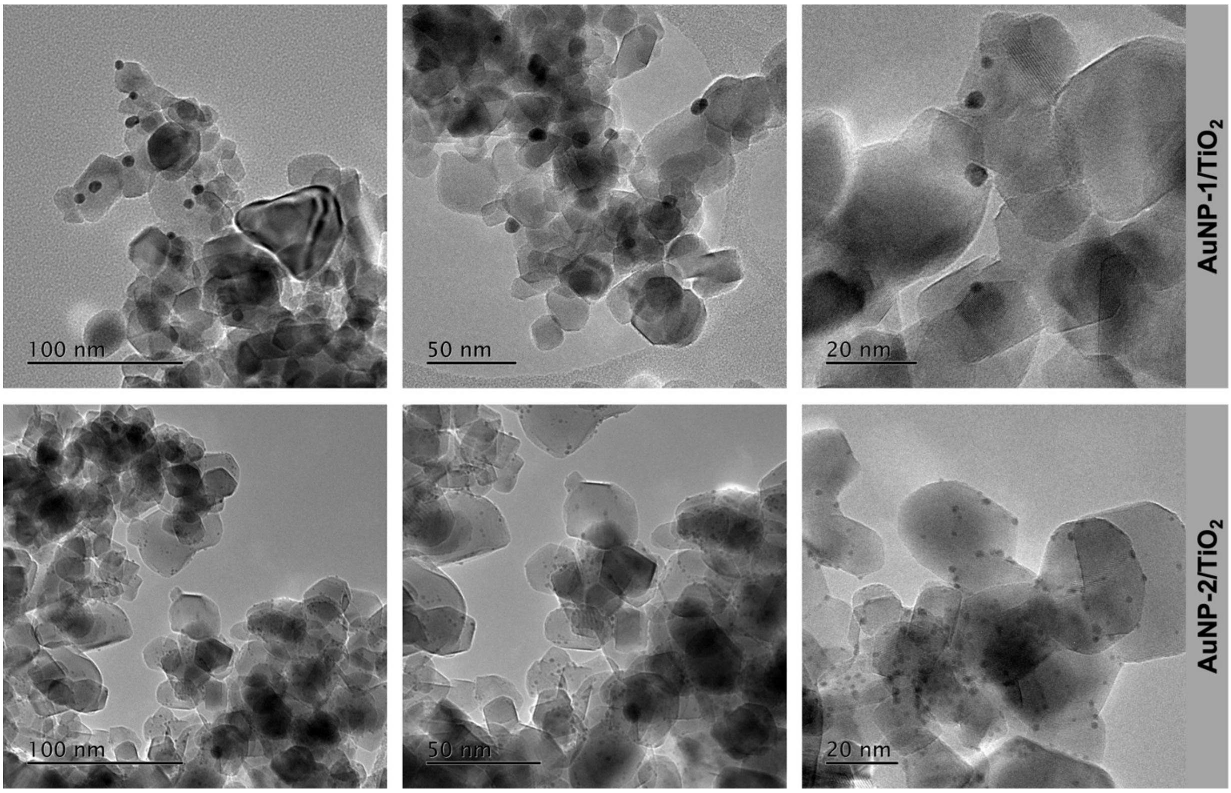

Figure S21. TEM images of **AuNP-1/TiO₂** (top) and **AuNP-2/TiO₂** (bottom) at different magnifications.

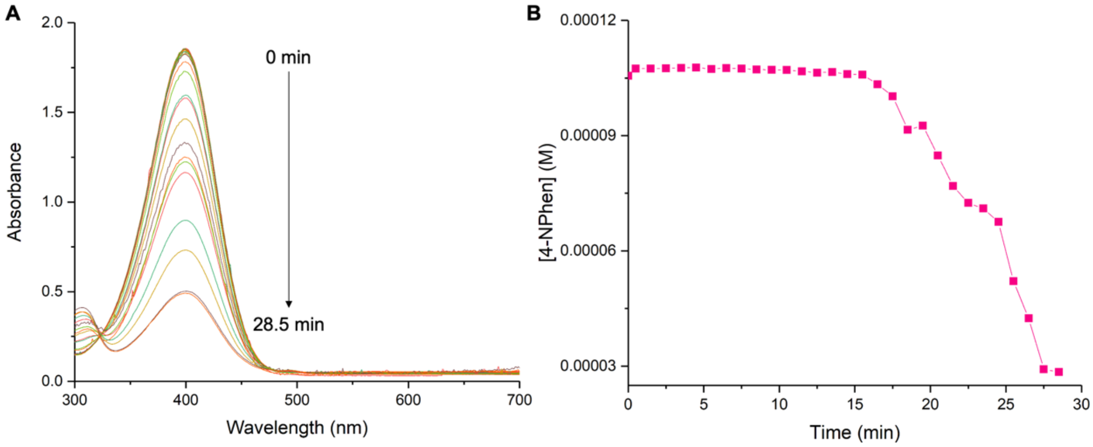

Figure S22. **A**) UV-Vis absorption kinetic study for the reduction of 4-nitrophenol (4-NPhen) into 4-aminophenol catalysed by **AuNP-1/TiO₂** (2.4 mg) in water at r.t. over 28.5 min, with a spectrum recorded every 30 sec. **B**) Plot of [4-NPhen] *vs.* time at 400 nm.

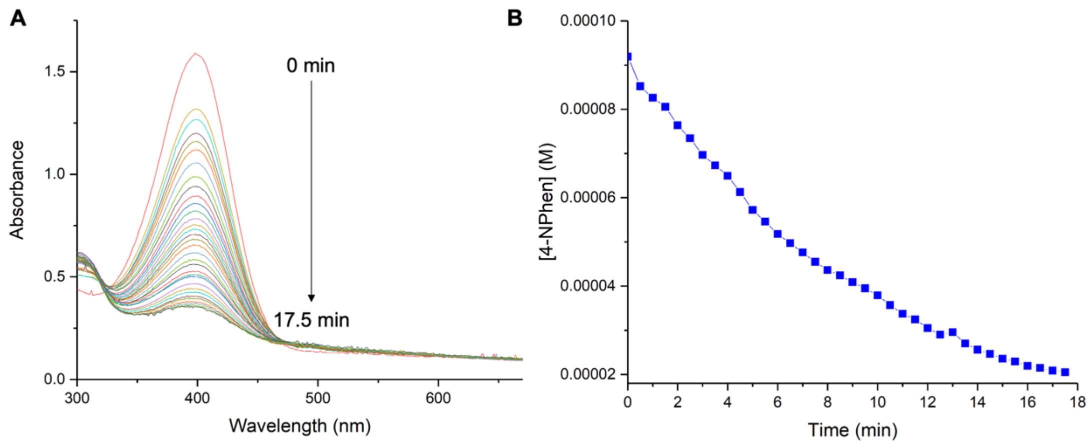

Figure S23. **A**) UV-Vis absorption kinetic study for the reduction of 4-nitrophenol (4-NPhen) into 4-aminophenol catalysed by **AuNP-2/TiO$_2$** (2.4 mg) in water and r.t. over 17.5 min, with a spectrum recorded every 30 sec. **B**) Plot of [4-NPhen] *vs.* time at 400 nm.

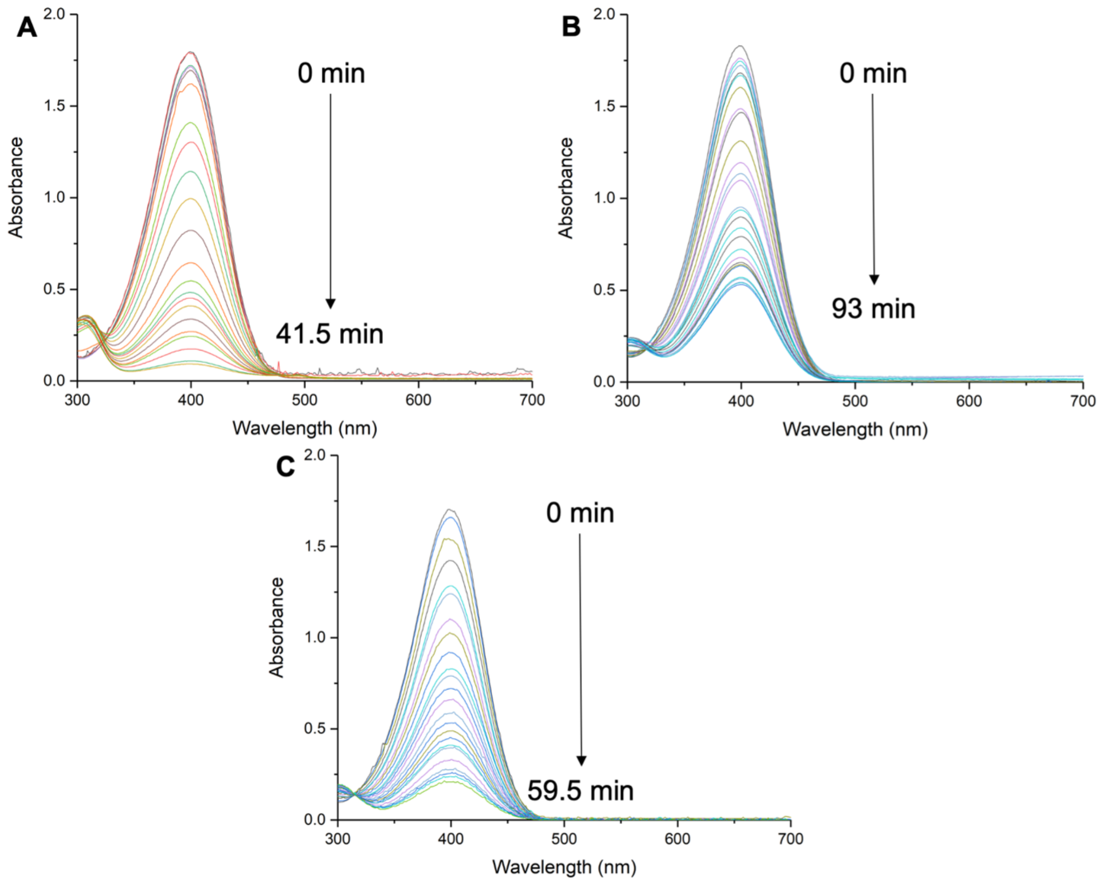

Figure S24. UV-Vis absorption kinetic study for the reduction of 4-nitrophenol (4-NPhen) into 4-aminophenol catalysed by **AuNP-1/TiO$_2$** (2.4 mg) in water and r.t. until maximum conversion reached, with a spectrum recorded every 30 sec over three cycles: **A**) cycle 1, **B**) cycle 2 and **C**) cycle 3.

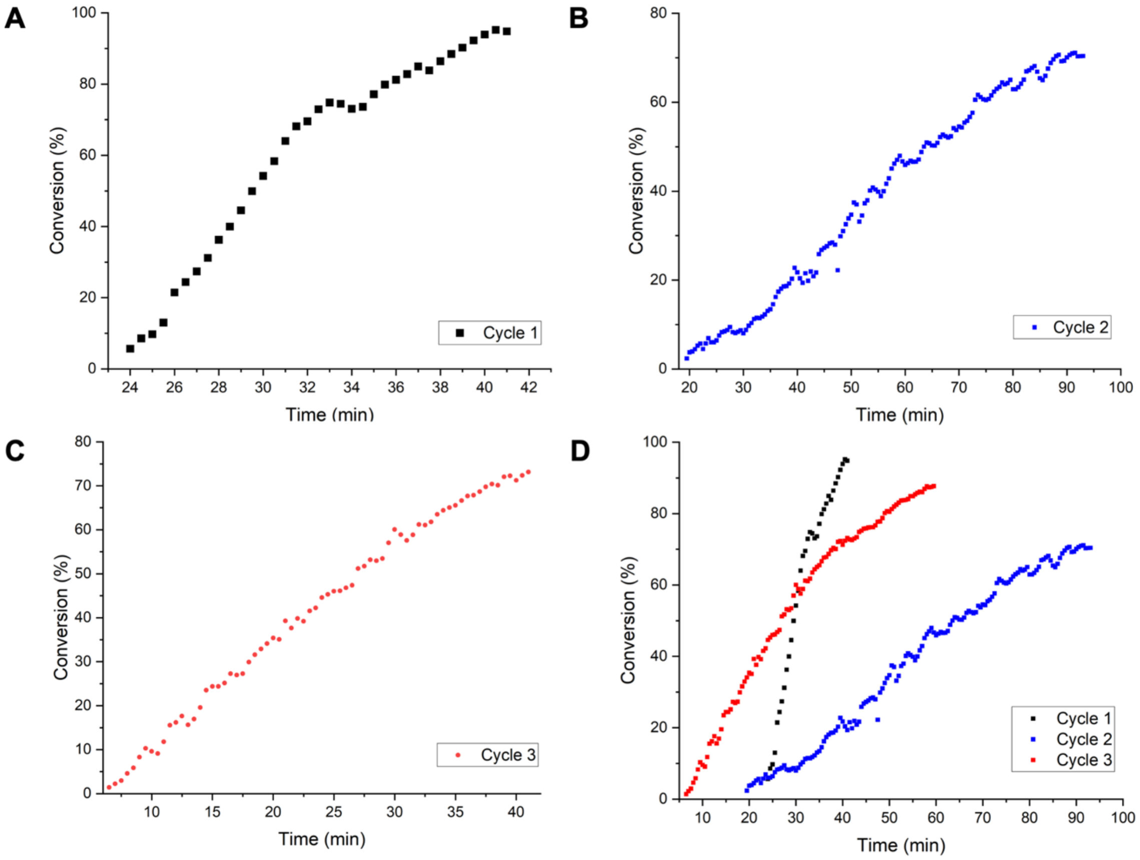

Figure S25. Plots of % substrate conversion *vs*. time for the reduction of 4-nitrophenol with **AuNP-1/TiO_2** over three cycles: **A**) cycle 1, **B**) cycle 2, **C**) cycle 3 and **D**) comparison of all three cycles.

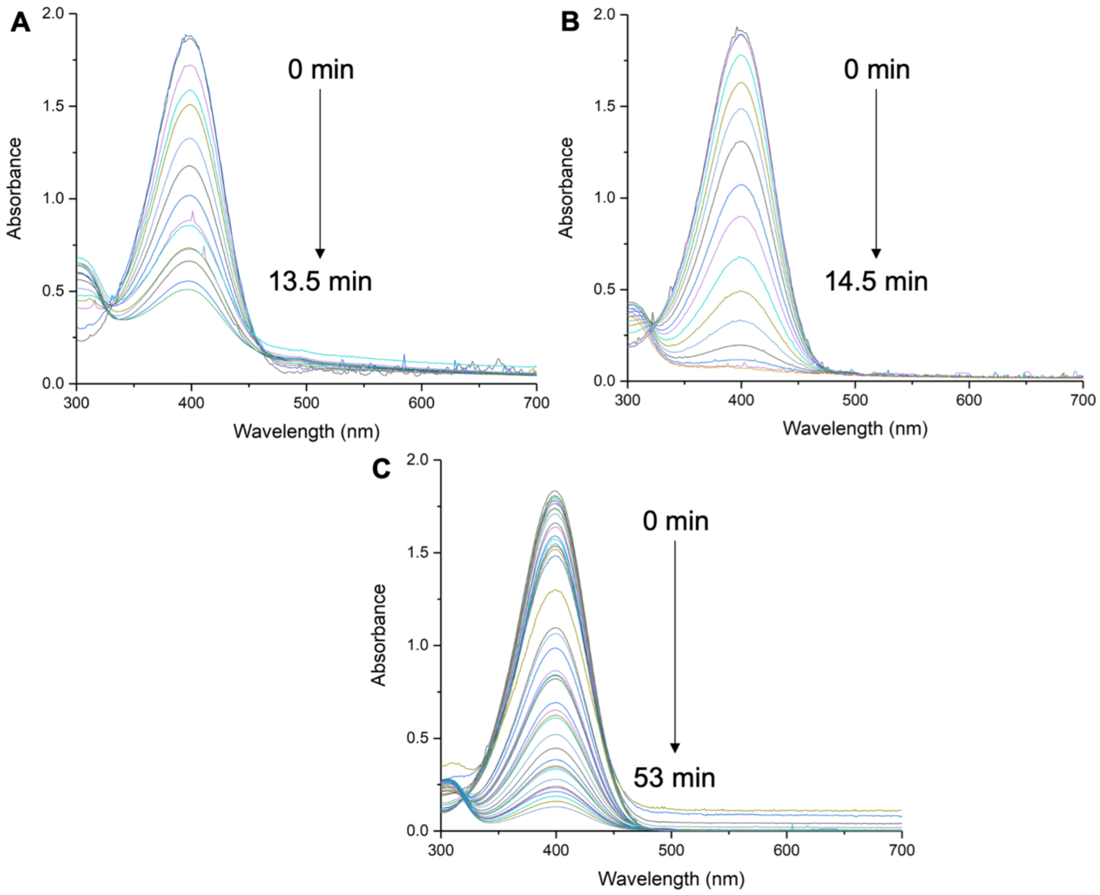

Figure S26. UV-Vis absorption kinetic study for the reduction of 4-nitrophenol (4-NPhen) into 4-aminophenol catalysed by **AuNP-2/TiO_2** (2.4 mg) in water and r.t. until maximum conversion reached, with a spectrum recorded every 30 sec over three cycles: **A**) cycle 1, **B**) cycle 2 and **C**) cycle 3.

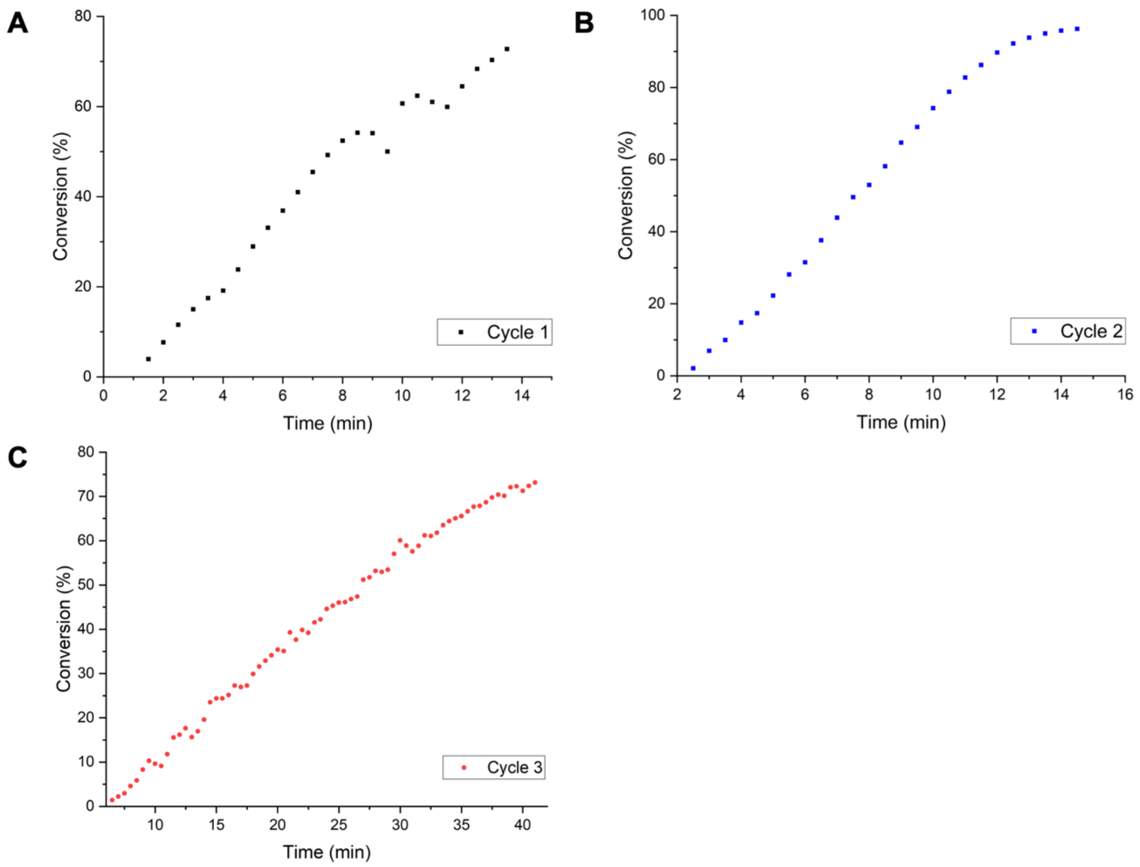

Figure S27. Plots of % substrate conversion *vs.* time for the reduction of 4-nitrophenol with **AuNP-2/TiO_2** over three cycles: **A**) cycle 1, **B**) cycle 2, **C**) cycle 3.

Tables

Table S1. XPS data (BE and composition) for **AuNP-1** and **AuNP-2**.

	**AuNP-1**		**AuNP-2**	
**Peak**	**BE (eV)**	**%At Conc.**	**BE (eV)**	**%At Conc.**
Au 4f7/2 (Au(I))	85.2	0.31	85.1	1.87
Au 4f7/2 (Au(0))	83.9	0.79	83.4	1.15
C 1s	284.8	61.53	284.9	39.6
C 1s	286.3	10.04	286.3	17.64
C 1s	288.7	6.18	288.3	3.51
N 1s	400.9	1.2	401.1	6.67
Na 1s			1072.0	0.92
O 1s	533.3	7.12	532.9	7.6
O 1s	531.9	11.97	531.6	15.91
S 2p (SOx)	168.1	0.87	167.8	5.13

Table S2. XPS data (BE and composition) for **AuNP-1/TiO_2** and **AuNP-2/TiO_2**.

	**AuNP-1/TiO_2**		**AuNP-2/TiO_2**	
**Peak**	**BE (eV)**	**%At Conc.**	**BE (eV)**	**%At Conc.**
Au 4f7/2 (Au(I))			85.0	0.05
Au 4f7/2 (Au(0))	82.9	0.11	83.4	0.77
C 1s	284.6	41.08	284.8	25.53
C 1s			286.2	5.57
C 1s			288.7	3.72
N 1s			400.2	0.88
O 1s (Ti-O)	529.5	31.65	529.7	36.33
O 1s	531.7	10.9	531.7	8.69
O 1s	533.2	1.64	533.5	0.99
S 2p (SOx)			167.8	0.55
Ti 2p	458.3	14.63	458.5	16.92

Table S3. XPS data (BE and composition) for **AuNP-1/TiO_2** before and after three 4-nitrophenol reduction reactions.

	Before Reaction		After Reactions	
Peak	BE (eV)	%At Conc.	BE (eV)	%At Conc.
Au 4f7/2 (Au(0))	82.9	0.06	82.7	0.08
C 1s	284.6	41.08	284.6	27.59
O 1s (Ti-O)	529.5	31.65	529.2	32.42
O 1s	531.7	10.9	531.3	15.6
O 1s	533.2	1.64	533.7	0.7
Ti 2p	458.3	14.63	458.0	15.12

Table S4. XPS data (BE and composition) for **AuNP-2/TiO_2** before and after three cycles of 4-nitrophenol reduction reactions.

	Before Reactions		After Reactions	
Peak	BE (eV)	%At Conc.	BE (eV)	%At Conc.
Au 4f7/2 (Au(I))	85.0	0.03		
Au 4f7/2 (Au(0))	83.4	0.44		
C 1s	284.8	25.53	284.8	35.74
C 1s	286.2	5.57		
C 1s	288.7	3.72		
Na 1s			1071.4	3.37
N 1s	400.2	0.88		
O 1s (Ti-O)	529.7	36.33	529.6	33.46
O 1s	531.7	8.69	531.8	11.51
O 1s	533.5	0.99	533.5	0.83
S 2p (SOx)	167.8	0.55		
Ti 2p	458.5	16.92	458.3	15.09

Table S5. Parameters used for photothermal conversion efficiency calculation of **AuNP-2**[a]

[AuNP-2] (mg/mL)	τ	hS	ΔT	A_{808}	Q_{Dis}	H (%)
0.4	290	14.51	6.3	0.63	26.37	25
0.2	316	13.27	4.5	0.32	26.37	18.8
0.1	278	15.11	3.1	0.16	26.37	19.5
Blank[b]	159	26.37	1	-	-	-

[a] τ (s) & hS (mW/°C) is calculated based on the photothermal effect of different samples and the equations 1-3 reported in the experimental section. Q_{Dis} is calculated based on the photothermal effect graph of DI water. $Q_{Dis\ water}$= hS*ΔT = 26.37 mW. I = 340 mW. [b] Blank sample containing PBS buffer (pH 7.4) only.
